# Climate change challenges, plant science solutions

**DOI:** 10.1093/plcell/koac303

**Published:** 2022-10-12

**Authors:** Nancy A Eckardt, Elizabeth A Ainsworth, Rajeev N Bahuguna, Martin R Broadley, Wolfgang Busch, Nicholas C Carpita, Gabriel Castrillo, Joanne Chory, Lee R DeHaan, Carlos M Duarte, Amelia Henry, S V Krishna Jagadish, Jane A Langdale, Andrew D B Leakey, James C Liao, Kuan-Jen Lu, Maureen C McCann, John K McKay, Damaris A Odeny, Eder Jorge de Oliveira, J Damien Platten, Ismail Rabbi, Ellen Youngsoo Rim, Pamela C Ronald, David E Salt, Alexandra M Shigenaga, Ertao Wang, Marnin Wolfe, Xiaowei Zhang

**Affiliations:** Senior Features Editor, *The Plant Cell*, American Society of Plant Biologists, USA; USDA ARS Global Change and Photosynthesis Research Unit, Urbana, Illinois 61801, USA; Centre for Advanced Studies on Climate Change, Dr Rajendra Prasad Central Agricultural University, Samastipur 848125, Bihar, India; School of Biosciences, University of Nottingham, Nottingham, NG7 2RD, UK; Rothamsted Research, West Common, Harpenden, Hertfordshire, AL5 2JQ, UK; Plant Molecular and Cellular Biology Laboratory, Salk Institute for Biological Studies, La Jolla, California 92037, USA; Biosciences Center, National Renewable Energy Laboratory, Golden, Colorado 80401, USA; School of Biosciences, University of Nottingham, Nottingham, NG7 2RD, UK; Future Food Beacon of Excellence, University of Nottingham, Nottingham, NG7 2RD, UK; Plant Molecular and Cellular Biology Laboratory, Salk Institute for Biological Studies, La Jolla, California 92037, USA; Howard Hughes Medical Institute, Salk Institute for Biological Studies, La Jolla, California 92037, USA; The Land Institute, Salina, Kansas, USA; Red Sea Research Center (RSRC) and Computational Bioscience Research Center, King Abdullah University of Science and Technology (KAUST), Thuwal, 23955-6900, Saudi Arabia; International Rice Research Institute, Rice Breeding Innovations Platform, Los Baños, Laguna 4031, Philippines; Department of Plant and Soil Science, Texas Tech University, Lubbock, Texas 79410, USA; Department of Biology, University of Oxford, Oxford, OX1 3RB, UK; Department of Plant Biology, Department of Crop Sciences, and Institute for Genomic Biology, University of Illinois at Urbana-Champaign, Illinois 61801, USA; Institute of Biological Chemistry, Academia Sinica, Taipei 11528, Taiwan; Institute of Biological Chemistry, Academia Sinica, Taipei 11528, Taiwan; Biosciences Center, National Renewable Energy Laboratory, Golden, Colorado 80401, USA; Department of Agricultural Biology, Colorado State University, Fort Collins, Colorado 80523, USA; The International Crops Research Institute for the Semi-Arid Tropics–Eastern and Southern Africa, Gigiri 39063-00623, Nairobi, Kenya; Embrapa Mandioca e Fruticultura, Rua da Embrapa, Cruz das Almas, BA, Brazil; International Rice Research Institute, Rice Breeding Innovations Platform, Los Baños, Laguna 4031, Philippines; International Institute of Tropical Agriculture (IITA), PMB 5320 Ibadan, Oyo, Nigeria; Department of Plant Pathology and the Genome Center, University of California, Davis, California 95616, USA; Department of Plant Pathology and the Genome Center, University of California, Davis, California 95616, USA; Innovative Genomics Institute, Berkeley, California 94704, USA; School of Biosciences, University of Nottingham, Nottingham, NG7 2RD, UK; Future Food Beacon of Excellence, University of Nottingham, Nottingham, NG7 2RD, UK; Department of Plant Pathology and the Genome Center, University of California, Davis, California 95616, USA; National Key Laboratory of Plant Molecular Genetics, Center for Excellence in Molecular Plant Sciences, Institute of Plant Physiology and Ecology, Chinese Academy of Sciences, Shanghai 200032, China; Auburn University, Dept. of Crop Soil and Environmental Sciences, College of Agriculture, Auburn, Alabama 36849, USA; National Key Laboratory of Plant Molecular Genetics, Center for Excellence in Molecular Plant Sciences, Institute of Plant Physiology and Ecology, Chinese Academy of Sciences, Shanghai 200032, China

## Abstract

Climate change is a defining challenge of the 21st century, and this decade is a critical time for action to mitigate the worst effects on human populations and ecosystems. Plant science can play an important role in developing crops with enhanced resilience to harsh conditions (e.g. heat, drought, salt stress, flooding, disease outbreaks) and engineering efficient carbon-capturing and carbon-sequestering plants. Here, we present examples of research being conducted in these areas and discuss challenges and open questions as a call to action for the plant science community.

## Teaser

We discuss research aimed at improving carbon sequestering capacity and climate resilience in plants to illustrate how plant science can help to mitigate climate change and enhance food security.

## Introduction

Climate change is caused by an accumulation of greenhouse gases (GHGs) (e.g. CO_2_, methane) in the atmosphere leading to increased planetary heat-trapping and global warming. The IPCC Sixth assessment report (IPCC, 2022) strongly suggests that limiting global warming to 1.5ºC above pre-industrial levels will be needed to avoid severe climate change effects. This will require halving global CO_2_ emissions by 2030 and cutting them to net zero by 2050, as well as removing an additional 2–10 billion metric tons (Gt) of CO_2_ each year. In some locations, warming may benefit certain crops, and, over time, the optimal growing regions may shift farther away from the equator. However, the effects of climate change are not limited to increasing temperatures and heatwaves in many parts of the world but include changes in rainfall, more severe and frequent storms, increased drought, and increased threat of wildfires. All of these effects are anticipated to adversely affect crop yields and food security worldwide within the next 20 years (Zhao et al., 2017; Li et al. 2019; Jägermeyr et al., 2021). As the impact of climate change on crop systems intensifies, the need to develop stress-resilient crops to combat food insecurity rises.

In this article, we explore several ways in which plant scientists are working on solutions related to carbon sequestration to help achieve net zero CO_2_ emissions and crop improvements to protect and enhance yields for increased food security. The first section outlines challenges and approaches for enhancing the carbon sequestration capacity of crops (annual and perennial) and seagrasses, followed by a section on improving photosynthesis. A third section addresses engineering climate resilience in crops (resistance or tolerance to abiotic and biotic stresses). The final section describes the vision of a sustainable global bioeconomy rooted in plant biology. We acknowledge that there are other areas, not covered here, in which plant science can play a role in mitigating adverse climate change effects, including bioenergy, forestry, and ecosystem conservation. Solutions in all of these areas are needed in the very near future, and in the longer term. We do not provide an in-depth review of these topics. Rather, the examples provided here illustrate a few of the many avenues of research being conducted by plant scientists around the world. A companion review by Verslues et al. (2023) addresses unresolved questions in plant abiotic stress. We hope that these stories help to inform the plant science community of the possibilities, stimulate further research, and motivate plant scientists at any stage of their careers to become involved in work aimed at mitigating climate change and enhancing food and energy security. Mitigating the climate change crisis will require all hands on deck.

## How can more carbon be retained in soil and biomass?

### Carbon sequestration in annual cropping systems

#### (By John K. McKay)

Annual cropping systems present opportunities for carbon sequestration that have yet to be exploited. In addition to the need to reduce GHG emissions, active atmospheric CO_2_ removal strategies, also called Negative Emissions Technologies (NETs), are needed to attain net CO_2_ reductions and avoid the most damaging climate change outcomes (National Academies of Sciences, Engineering, and Medicine, 2019). Atmospheric CO_2_ removal technologies need to be implemented now and increase to levels on the order of 10 Gt CO_2_ per year by 2050, and 20 Gt CO_2_ per year by 2100 (National Academies of Sciences, Engineering, and Medicine, 2019).

Among NET for CO_2_ removal, soil carbon sequestration is the least expensive and most ready to scale in the next decades (National Academies of Sciences, Engineering, and Medicine, 2019). Current US cropping systems use genetics that were not designed to minimize GHG emissions nor to maximize carbon sequestration, yet heritable genetic variation for these traits exists in many crops. In addition, agricultural soils experienced well-documented decreases in soil carbon over the last century (Davidson and Ackerman, 1993) and are capable of sequestering all of the CO_2_ currently in the atmosphere (Ciais et al., 2013). Here, I review the challenges with attempts to achieve soil carbon sequestration in current annual cropping systems, both with the way in which the maize (*Zea mays*)–soy (*Glycine max*) rotation was designed and the science to date on how management might lead to predictable increases in soil carbon. I then focus on genetic changes that are needed to create carbon-negative crops, including optimal combinations of traits that can be addressed in breeding programs.

The major, unaddressed problem for sustainability and GHG emissions in annual cropping systems is excess nitrogen (N) in the form of synthetic fertilizer (Northrup et al., 2021), which leaches into groundwater, rivers, and oceans and into the atmosphere as N_2_O, a GHG with an effect size ∼300× that of CO_2_ (Albritton et al., 2001). An obvious example is ethanol production from maize, where N is responsible for >80% of GHG emissions overall (Kim et al., 2014). For the parts of the world where the Green Revolution was successfully deployed (Evenson and Gollin, 2003), a major consequence is the exclusive use of crop genotypes that require high N inputs. To fix this N problem, we need to improve N use efficiency (NUE) and greatly reduce N input. Increasing NUE is feasible (Hirel et al., 2007; Northrup et al., 2021) and can be achieved in part by removing a small number of large-effect mutations that were selected to high frequency in elite germplasm in the Green Revolution (Moyers et al., 2018). Getting farmers to reduce N input is a much greater challenge. First, overfertilizing every other year is a well-established management practice of the maize-soy rotation that encompasses 73 million hectares of farmland in the USA. Although soybean is an N-fixing species, in modern cropping systems high-yielding soy crops require hundreds of kilograms of N per hectare (Salvagiotti et al., 2008). Although fertilizer has recently increased in price, so have crop commodity prices, and thus farmers remain incentivized to maximize N inputs. In the USA, the maize–soy rotation is highly subsidized by federal funds in the form of direct payment to farmers as well as mandates on using ethanol from fermentation of maize grain and biodiesel from transesterification of soy lipids.

Most efforts in using annual cropping systems for soil carbon sequestration have focused on changes in management that were originally designed for soil health (Ogle et al., 2019), such as reduced tillage, greater residue retention, and cover crops that are designed to increase the amount of above-ground plant biomass left in the field per unit area per year (McClelland et al., 2021). Most of the published studies on the effect of management on soil carbon are limited to the top 30 cm of soil, which is where most of the carbon inputs are expected (Ogle et al., 2019). However, this top 30 cm is also the least durable soil carbon and can respire back into CO_2_ in a few years. Getting soil carbon inputs deeper into the soil is needed to achieve greater and more durable carbon sequestration in agricultural systems (Paustian et al., 2016a, 2016b, 2019) and will require genetic changes in crops.

Genetic changes in annual cropping systems are needed both to reduce inputs (Northrup et al., 2021) and achieve carbon sequestration levels of tons per hectare per year (Paustian et al., 2016a). Some changes can be achieved by selecting against large-effect mutations that went to high frequency during the Green Revolution. Prior to the Green Revolution, putting large amounts of synthetic N on agricultural fields reduced yield, as tall crops heavy with grain were highly prone to lodging. In many cases selection during the Green Revolution was based on recurrent backcrossing to dwarf lines and involved small effective population sizes and low levels of effective recombination (Moyers et al., 2018). For example, in rice (*Oryza sativa*), breeding during the Green Revolution led to the fixation of mutations that reduce NUE (OsTCP19; Liu et al., 2021b) and root growth (Dro1; Arai-Sanoh et al., 2014) in the elite breeding lines.

**Figure 1:**
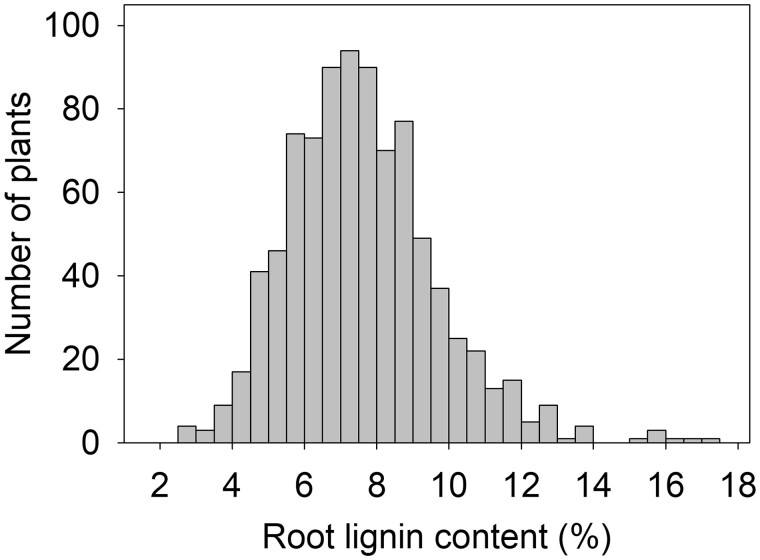
Quantitative variation in lignin content in maize root systems from a field study of 358 maize inbred lines. A description of the experiment can be found in Woods et al. (2022).

It is worth considering the traits of an ideal annual crop for carbon-negative supply chains for food, feed, fiber, and fuel. As mentioned, genetic changes to lower N requirements and create deeper, more massive root systems can make annual biomass feedstock production carbon negative (Paustian et al., 2016a). Another key trait for carbon sequestration is population density, where increasing the number of individuals per hectare leads to more root systems and greater carbon input. Crop species that were not part of the Green Revolution have promise in this respect (Amaducci et al., 2008). For example, industrial hemp (*Cannabis sativa*) was never bred for high N inputs, can be grown at population densities of 500,000 plants per hectare, and has greater root biomass below 50 cm than other major crops (Amaducci et al., 2008). Root carbon composition is also a genetic target, as some forms of carbon may be more recalcitrant to degradation and therefore longer lived in soils. The idea of engineering roots to create more recalcitrant forms of carbon, such as suberin, is discussed below by Busch and Chory. Suberin is one example; another is lignin, which is a parameter in models of soil carbon (Parton, 1996). We found large heritable variation in percent lignin in maize roots (Figure 1) and are testing the prediction that genotypes with greater root lignin will lead to greater quantity and durability of soil carbon.

Root exudates, a diverse set of simple carbon molecules that are released passively or actively into the soil, also contribute to soil organic carbon (SOC). Little is known regarding the degree to which root exudates are controlled by genetics versus the environment. Even less is known about the genetic control of the abundance and composition of root exudates, even in model species. This is due in part to the difficulty of measuring the relevant phenotypes in agriculturally relevant environments. On the soil modeling side, recent progress has been made in separating biomass and exudate inputs to soil carbon, where exudates are predicted to lead to increases in mineral-associated organic matter, which in turn is predicted to have a longer residence time than other soil carbon fractions (Zhang et al., 2021b). Finally, the soil and root microbiome, which is influenced by root exudates and plant genotype (Peiffer et al., 2013; Wagner et al., 2020; Favela et al., 2021), influences the carbon retention properties of soils, although data on effect sizes are lacking (Naylor et al., 2020). Ectomycorrhizae are thought to be key drivers of SOC accumulation in forests (Soudzilovskaia et al., 2019) and could be exploited in cropping systems. Manipulating the soil and root microbiome of cropping systems at scale will be much more difficult than obtaining seed from new crop genotypes but is a possible tool for engineering annual cropping systems for enhanced carbon sequestration.

### Harnessing plants: A global initiative to enhance plant-based carbon sequestration

#### (By Wolfgang Busch and Joanne Chory)

We consider solutions for carbon sequestration based on plants’ abilities to draw down CO_2_ from the atmosphere via photosynthesis and convert it to biomass. Earth’s soils contain a large amount of carbon, estimated at approximately 2,300 Gt carbon to 3-m depth, which constitutes about three times the current atmospheric pool of CO_2_ (Schlesinger and Bernhardt, 2020). The main source of SOC is plant material (e.g. aboveground plant biomass, roots, and root exudates), which can be stored in the soil or respired back into the atmosphere. It is estimated that cropland and grazing land soils (about 5 billion hectares globally) have an enormous capacity for storing carbon (Sanderman et al., 2017). Combined with existing agricultural infrastructure, this capacity provides an opportunity to leverage genetics to improve traits related to plant-mediated carbon sequestration.

**Figure 2:**
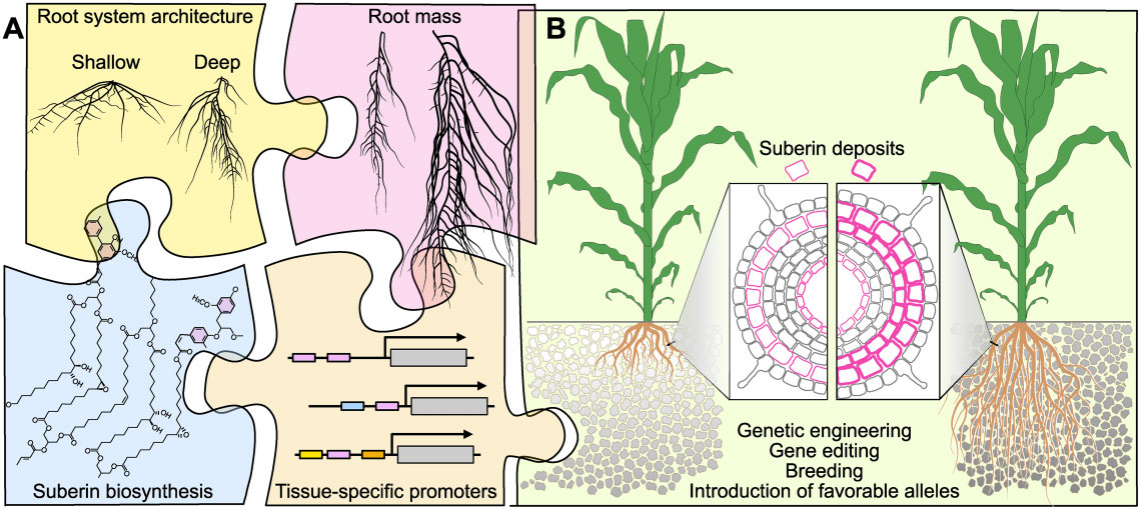
Toward an ideal carbon-capturing crop plant. A, The ideal plant should accumulate suberin in the cell wall of its root cells and form a vast and deep root system. To realize this goal, the existing literature and experimental evidence are curated to look for candidate genes affecting root system architecture and root mass. This information is combined with root-specific promoters and suberin biosynthetic genes. B, The ideal plant is created by capitalizing on both classical (breeding) and more recent (genome editing, genetic engineering) approaches to introduce favorable alleles and genes that will increase root biomass and transgenes that will increase the deposition of suberin in the root. In addition to trapping more carbon, these ideal plants will replenish carbon-depleted soils with degradation-recalcitrant carbon polymers (indicated by the darker color of the soil on the right). Figure credit: P. Salomé.

Several plant traits are good candidates for facilitating plant carbon sequestration (Figure 2). Root biomass is one, as it is estimated that a given mass of root inputs contributes about five times more SOC than the equivalent mass of aboveground litter (Jackson et al., 2017). However, traits associated with mechanisms that increase recalcitrance of SOC to breakdown by soil microorganisms (SOC protection) will also be required to increase residence time in soils. Mechanisms of SOC protection include a complex interplay between the chemical makeup of SOC, physical occlusion of SOC within soil aggregates, formation of stable organo-mineral complexes, and water-film connectivity between SOC and microbes (Schmidt et al., 2011; Lehmann et al., 2020). More than half of the global SOC is found in deep soil layers (Jobbágy and Jackson, 2000), and the mean residence time of SOC increases with depth, implying lower decomposability of root-derived carbon in deeper soil layers (Gill et al., 1999; Prieto et al., 2016). Root biochemistry also influences decomposability, and a prime candidate trait is the amount of the natural product suberin in roots. Suberin is a lipophilic complex polyester that is composed of very long-chain fatty acids and polyaromatic compounds. Suberin may be a good source for stable SOC due to its intrinsic biochemical stability (Lorenz et al., 2007) and its interaction with soil minerals and occlusion in topsoil microaggregates (Kell, 2012; Lin and Simpson, 2016). We note that there are numerous other plant traits that promise to be useful for enhancing the capacity of plants to sequester carbon in the soil (see some additional examples in the previous section by McKay).

There are several significant challenges to utilizing crops for carbon sequestration. Genetic trait enhancement is a lengthy process and its adoption by the public will be challenging. Establishing a link between root traits and carbon accumulation and permanence in agricultural soils will require substantial experimental efforts. Carbon accumulation and persistence are also dependent on soil type, climate parameters, and agricultural practices such as the use of cover crops and no-till farming (Schmidt et al., 2018). Although there is good potential for plant-based carbon sequestration in the surface soil layer (up to 1.85 Gt C/year in the top 30 cm of global cropland soils alone; Zomer et al., 2017), an enhanced rooting depth and altered biochemical makeup of roots could yield a much larger sequestration capacity. Finally, time is pressing—every year that goes by without significant carbon drawdown will negatively impact billions of humans and decrease the biodiversity of our planet.

The Salk Harnessing Plants Initiative is working to identify genetic and molecular mechanisms to increase root biomass, root depth, and suberin root content. We use examples of this research to highlight considerations for plant-based carbon sequestration that we have identified during this work. Each of the target traits comes with specific challenges and opportunities. For instance, increased root mass will elevate the carbon input into soils and can improve the ability of roots to forage for nutrients and water. However, increasing root mass beyond a certain level might come at the expense of yield. Nevertheless, the relationship between root biomass and yield is not necessarily a zero-sum game as enhanced water and nutrient uptake of a bigger root system can support a larger shoot. This might be particularly relevant under drought or nutrient-limited conditions. An example of the lack of a strict tradeoff of root biomass and yield in major crops is the lack of correlation of yield and root biomass in maize as well as soybean in a multi-location, multi-year study (Ordóñez et al., 2020). Increasing root depth promises to increase the lifetime of the average carbon molecule deposited by roots in the soil, provide roots access to deeper soil layers that can contain more moisture, and facilitate the capture of nitrate that leaches deeper into the soil during the growing season. However, surface roots are still important for foraging immobile nutrients such as phosphorus. Therefore, achieving an optimal balance between shallow roots and deep roots will be important. As an effective apoplastic barrier, suberin in specific areas of the root could provide enhanced flood and drought resilience and might enhance root growth in deeper, more anoxic layers of the soil. Extensive variation for each of these traits between and within species indicates that there are genetic mechanisms that can be leveraged to improve them.

Our work in enhancing these traits is being conducted in parallel with model plants via forward and reverse genetic approaches, as well as in diversity collections of major row crops and cover crops to identify crop-specific targets using genome-wide association studies (GWASs). While we are interested in trait changes that will work in the field and maintain crop productivity, it is not feasible to measure all these root traits in the field at high throughput. We therefore rely on initial screening approaches in the laboratory or the greenhouse to measure and engineer root traits, subsequently moving to in-soil or field-testing with a subset of lines that display distinctive traits. We focus on root mass in relation to depth, as the engineering or breeding goal is to direct as much root mass as possible to a deeper depth, and on enhancing the accumulation of suberin. Suberin is a highly effective apoplastic diffusion barrier and producing it everywhere in the root would be detrimental to plant health. Therefore, we focus on specific root tissues that already produce suberin such as the periderm or the exodermis, which are outer layers in mature root systems. We are targeting such tissues as suberin sinks by using tissue-specific promoters to drive suberin production, as well as utilizing genes involved in the formation of these tissues to produce additional tissue layers.

To quantitatively link these root traits to carbon characteristics in the soil, we are working with soil scientists to better estimate the soil carbon impact of crop varieties that have different root mass, depth, and suberin content. We aim to test the effects of genetic alterations via gene editing or gene engineering approaches in crops over the next few years. Recent advances in high-throughput phenotyping, sequencing, and functional single-cell genomics now provide a way to leverage genes, gene constructs, and genetic variants within and between species. We aim to have the proof of concepts for enhanced crop traits within the next 3 years to then partner with both NGOs and agriculture companies to enhance varieties that are of interest to farmers.

There are numerous other opportunities for plant biologists to contribute to climate change mitigation efforts, ranging from work on traits that will reduce agricultural N_2_O or methane emissions to creating carbon sequestration-friendly microbiota or mycorrhizal associations. As a community, we should think of and work toward promising plant biology-based solutions.

### Rapid de novo domestication of perennial crops

#### (By Lee R. DeHaan)

Most agricultural soils have lost 50%–70% of the SOC that they had previously accumulated under native plant communities; therefore, raising the carbon levels in historically tilled agricultural soils offers the potential to partially mitigate climate change by capturing 30–60 Gt of organic carbon (Lal, 2003). The restoration of SOC in agricultural soils would not only mitigate climate change through sequestration but would also contribute to adaptation to climate change by developing soils with greater nutrient holding capacity, resistance to erosion from extreme rain events, increased water infiltration, and water storage to stabilize productivity in the face of erratic rainfall (Blanco-Canqui et al., 2013).

Although planting long-lived perennial plants on degraded agricultural soils would be one of the most effective ways to rapidly restore soil carbon levels, this approach is limited because the herbaceous perennials currently available for use in agriculture (mainly forage crops) produce biomass that is unsuitable for direct human consumption (Paustian et al., 2016a). Therefore, efforts are underway to develop new crop plants that would have extensive long-lived root systems and would achieve carbon sequestration levels similar to perennial biofuels (Crews and Rumsey, 2017; Dheri et al., 2022) while simultaneously producing abundant human-edible protein, starch, and oils through mechanically harvestable grain (Glover et al., 2010).

Efforts to develop perennial grain crops began decades ago, but recent advances in genetics and breeding are accelerating the timeline and the first successful perennial grains are now entering fields and markets. A perennial rice breeding program was initiated in 1996, targeting the roughly 19 million hectares of upland rice grown worldwide where forest land is often cleared and degraded (Sacks et al., 2003). Annual rice (*Oryza sativa* ssp. *indica*) and the rhizomatous perennial relative *Oryza longistaminata* were hybridized, and a breeding program has produced lines for flooded paddies that persist through eight harvests with yields and quality traits on par with modern rice cultivars (Huang et al., 2018; Hu et al., 2022). Perennial paddy rice is expected to reduce GHG emissions and water consumption relative to annual rice (Oda et al., 2019). The development of perennial rice for upland conditions also remains possible in the near term.

**Figure 3:**
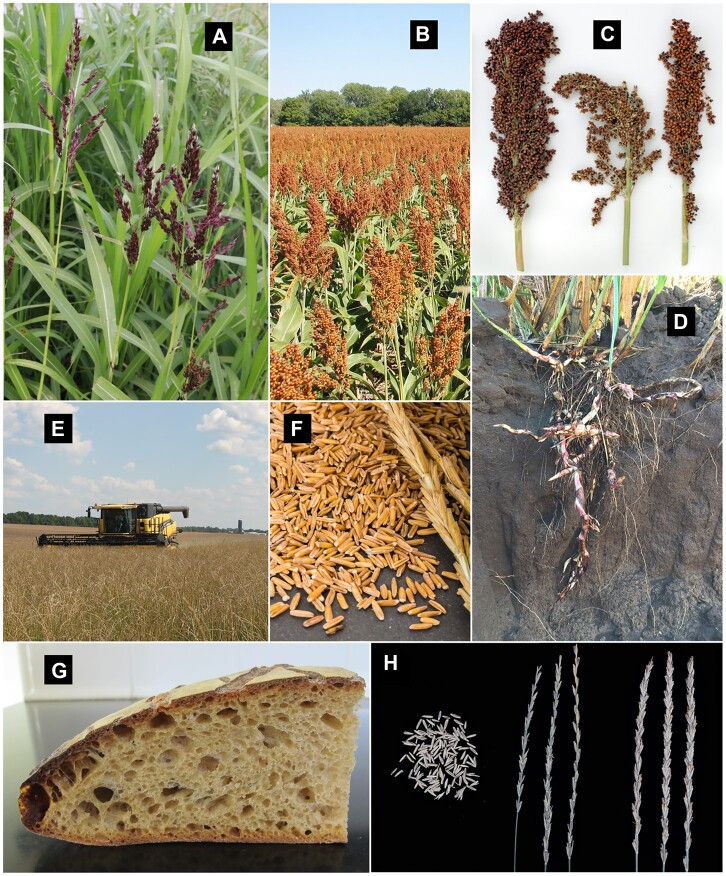
Examples of wide hybridization and direct domestication to develop perennial grains. The wild perennial *Sorghum halepense* (A) was hybridized with the domestic species *Sorghum bicolor* (B) and selective breeding of the progeny produced lines with intermediate head and seed size (C) and the ability to regrow from underground rhizomes (D). In an example of direct domestication, the mostly wild grass *Thinopyrum intermedium* can be harvested with conventional equipment (E) and cleaned to obtain a human-edible grain (F) that has properties similar to wheat, as seen in this loaf made with an 80/20 blend of wheat and *Th. intermedium* flour (G). Domesticated *Th. intermedium* types now possess domestication traits, such as shatter resistance (H, at right).

Perennial grain sorghum is being developed through wide hybridization of annual grain sorghum (*Sorghum bicolor*) with perennial species (Figure 3). Progress for yield and survival has been made by selecting among progeny of crosses between *S. bicolor* and the tetraploid perennial *Sorghum halepense*, and evaluation under tropical conditions suggests no barrier to high-yielding perennial varieties in warmer regions (Cox et al., 2018b). Recently, diploid perennial grain sorghum lines have been derived from diploid × tetraploid crosses (Cox et al., 2018a) and from crosses between *S. bicolor* and the perennial diploid species *Sorghum propinquum* (Foster et al., 2020). Working at the diploid level is expected to expedite the development of perennial grain sorghum by simplifying crosses between perennial germplasm and locally adapted *S. bicolor* varieties. Now, marker-assisted selection is being initiated to accelerate progress in breeding for traits related to perenniality and productivity (Cox et al., 2018b).

A wide array of perennial grain crops could likely be developed either by direct domestication of wild perennial species or wide hybridization between crops and related perennials. Perennial wheat with potential to improve soil quality (Audu et al., 2022) is being developed through wide hybridization (Hayes et al., 2018). A direct domestication program is underway to develop the perennial sunflower relative *Silphium integrifolium* into a dual-purpose forage and grain crop (Van Tassel et al., 2017). Various perennial leguminous species are also being considered for their suitability for use as perennial grains (Schlautman et al., 2018). Perennial flax (*Linum*) species are being evaluated for direct domestication as perennial oilseeds (Tork et al., 2019).

Direct domestication of the cool season perennial grass species intermediate wheatgrass (*Thinopyrum intermedium*; Figure 3) was initiated in the 1980s, and now the harvested grain is being produced and sold in North America under the trade name Kernza (DeHaan and Ismail, 2017). With its extensive root system (Sprunger et al., 2019), the crop has potential for carbon storage belowground (De Oliveira et al., 2020) and to accumulate microbial necromass (Peixoto et al., 2020). However, genetic improvement for grain yield is needed, since selected populations still have a yield potential of less than half that of bread wheat (*Triticum aestivum*) in the same region (Culman et al., 2013). In addition to the currently limited genetic potential for grain yield, the crop faces many challenges. New crops always struggle with the need to coordinate supply chain development in concert with expanding acreage. Novel perennial grains also introduce a new array of challenges for farmers and agronomists, such as controlling pests and diseases and managing for sustained yield over many years. With intermediate wheatgrass, the decline in yield that occurs in aging stands is an ongoing challenge (Pinto et al., 2021).

Recent developments in plant biology, genetics, and breeding have opened the door to breeding new crops with carbon-storing perennial root systems and abundant grain production at a time scale that can proceed at the pace of commercial enterprise development (Runck et al., 2014). Low-cost genome sequencing and innovative genome assembly approaches are allowing the rapid generation of reference sequences even for large-genome perennial species. Genomic information is now leveraged to perform genomic selection (Meuwissen et al., 2001) which can greatly accelerate the breeding of perennial crops. Whereas traditional breeding of a perennial crop might require 5 or more years per generation, involving field evaluation of multiple years followed by intermating of selected individuals, genomic selection uses a genomic prediction model based on the performance of plants grown from multiple generations over many years. Applying genomic models to genetic marker data from seedlings of intermediate wheatgrass has accurately predicted mature plant performance (Crain et al., 2021). Speed breeding (Watson et al., 2018) paired with genomic selection has the potential to further accelerate perennial crop improvement by increasing the number of generations that can be completed per year. Genomic selection with speed breeding is currently being implemented with intermediate wheatgrass to complete two full cycles of selection per year, compared to one cycle every 3 years with classical approaches. Although these methods hold great promise, they remain to be validated across the many repeated cycles of selection necessary to produce a highly productive domestic crop.

The application of genome editing techniques to the domestication of wild species creates exciting possibilities to compress the development timeline for new crops (Zsögön et al., 2018). For instance, by comparing the genome sequence of the perennial *Thinopyrum intermedium* with related domestic grains, targets for genome editing to obtain domestic phenotypes have been identified and a roadmap for rapid domestication established (DeHaan et al., 2020).

Although new crop development is the primary approach being used to produce new perennial grains, the rediscovery of “orphan” perennial grain species is another approach worthy of investigation. Pigeonpea (*Cajanus cajan*) is an N-fixing semi-perennial shrub that is grown in Asia and southern Africa. Although types that can be grown for several seasons without replanting have been used in erosion control and are still grown by some farmers, annual pigeonpea is now the dominant form. A recent study in Malawi indicated that farmers are less likely to adopt erratically performing perennial pigeonpea due to social pressures and lack of trust in the technology (Grabowski et al., 2019). Expanded acreage of soil-conserving perennial pigeonpea may depend on the development of improved management techniques spread through peer learning, and new cultivars that enable consistent production.

The basic genetic and physiological control of the perennial growth habit has only recently been the subject of experimentation and remains poorly understood (Park et al., 2017). This lack of understanding has thus far hindered the development of perennial grain crops. With a clear understanding of the pathways involved, rapid conversion of existing annual crops into perennials could be possible. Combined approaches of wide hybridization, genome editing, mutagenesis, and transgenics could be used to achieve perennial growth in high-yielding cultivars. Because the need for carbon sequestration in soils is urgent, these approaches could be implemented in parallel, following the approach used to develop COVID vaccines (Ball, 2020) to develop an array of high-yielding perennial crops in the coming years.

### The promise of seagrasses for carbon capture and storage

#### (By Carlos M. Duarte)

Seagrasses are a group of about 74 angiosperm species that complete their life cycle in the marine environment, where they form lush meadows that rank amongst the world’s most productive ecosystems (Duarte and Chiscano, 1999; Hemminga and Duarte, 2000). Seagrass meadows are strongly autotrophic, producing more organic matter than consumed in the ecosystem (Duarte et al., 2010) and acting, therefore, as sinks for CO_2_, much of which is buried in seagrass soils (Duarte et al., 2005, 2013a; Fourqurean et al., 2012). The role of seagrasses as intense carbon sinks in the biosphere is supported by their high photosynthetic efficiency, low nutrient requirements, adaptations that minimize carbon losses, and their capacity to cope with anoxic, sulfide-rich sediments. Indeed, whereas seagrasses occupy an estimated 0.08% of the ocean seafloor, they contribute an estimated 12.7% of all organic carbon annually buried in the ocean seafloor (Duarte et al., 2005). Yet at least one-third of the historical global area occupied by seagrasses has been lost, leading to the loss of this carbon sink and the risk of remineralization and subsequent CO_2_ emission of the carbon stocks accumulated in their soils over millennia. Hence, seagrass meadows represent a key component of the so-called “blue carbon” strategies aimed at avoiding losses and restoring coastal vegetated habitats to contribute to climate change mitigation, through carbon capture and storage, and climate change adaptation through the coastal protection seagrasses offer (Duarte et al., 2013a; Macreadie et al., 2021).

A range of tools within plant sciences, from genomics and metabolomics to microbiome investigations are providing important insights into the underpinnings of the remarkable carbon capture capacity of seagrass. Whereas the role of seagrasses in carbon capture and storage has been addressed largely through the quantification of stocks (Fourqurean et al., 2012) and burial rates (Duarte et al., 2005, 2013a), seagrass traits related to carbon capture and storage have been poorly addressed. Here, I discuss the fundamental plant traits that render seagrasses so efficient in carbon removal and identify a number of promising areas where further research may provide additional insights on their role. Further efforts in resolving carbon concentration mechanisms and the role of the microbiome, specifically the root component, offer promise to contribute to developments in carbon capture technologies and to increase the efficiency of seagrass restoration, respectively.

#### What do we know?

The high productivity of seagrass meadows even under low light conditions (Duarte and Chiscano, 1999) supplies much of the carbon sequestered in seagrass meadows (Kennedy et al., 2010). The keys to the high productivity of seagrass meadows are efficient light use (Enríquez et al., 1994), low nutrient requirements (Duarte, 1990), and carbon concentrating mechanisms that allow seagrasses to use both CO_2_ and ${\text{HCO}}_{3}^{-}$ to support their high photosynthetic rates (Larkum et al., 2006). The analysis of the full genome sequence of the seagrass *Zostera marina* pointed to a number of evolutionary adaptations required for these species to colonize the ocean from freshwater angiosperm ancestors (Olsen et al., 2016). Some of these adaptations help explain their high carbon removal, including the loss of volatiles, consistent with the loss of stomata through which they are emitted for airborne communication and plant defense, which reduces losses of carbon and the probability of infections, as stomata are a main entry point for pests and pathogens in terrestrial plants (Olsen et al., 2016).

The seagrass genome also revealed new combinations of structural traits related to the cell wall, enabling the synthesis of cutin-cuticular waxes, suberin–lignin near the plasma membrane, and macroalgal-like sulfated polysaccharides (Olsen et al., 2016), recently confirmed by direct analyses of the seagrass cell walls, which revealed the presence of fucose-containing sulfated polysaccharides, apiogalacturonan and lignin (particularly in roots and rhizomes; Pfeifer et al., 2022). This composition, together with low N and phosphorus content, renders seagrass tissues highly recalcitrant to microbial degradation (Enríquez et al., 1993), helping to explain high seagrass-derived lignin concentrations in seagrass soils (Nakakuni et al., 2021) and the high organic carbon preservation supporting high carbon sequestration rates. The full genome sequence conducted to date excluded endophytic prokaryotes (Olsen et al., 2016), which also have important contributions, as exemplified by the recent discovery of a symbiosis with an N-fixing, root-endophytic bacteria, which helps explain the high productivity of seagrass in oligotrophic environments (Mohr et al., 2021).

Seagrass morphology is a basic underpinning of their role in carbon removal. They are able to form dense canopies, exceeding 15 m^2^ of leaf surface per m^2^ of ground covered (Romero et al., 2006), and their rhizomes and roots also form a dense web in the sediments, with 0.18–3 m^2^ of rhizome per squaremeter and 0.47–1 m^2^ of roots per squaremeter of soil (Duarte et al., 1998). The dense web of seagrass leaves acts as a filter that retains particles entrained in the flow and dissipates wave and turbulent energy, enhancing the deposition and retention of particles in their soils (Hendriks et al., 2008). Meanwhile, the dense web of rhizomes and roots in the sediments injects a significant fraction of seagrass net production (2.8%–48.6% of total net production; Duarte et al., 1998) into the soil and provides physical cohesion, thereby reinforcing the soils against the erosive force of storms and extreme-energy events, such as tsunamis (Chatenoux and Peduzzi, 2007; Sasa et al., 2012).

Rhizome growth and meristematic dominance are the keys to the exponential clonal growth of seagrasses, which is a major driver of the efficiency of seagrass restoration projects in restoring seagrass carbon removal (Duarte et al., 2013b), as demonstrated in assessments of the carbon removal benefits of seagrass restoration (Marbà et al., 2015; Oreska et al., 2020). Seagrass restoration traditionally was small in scale and relatively expensive and inefficient, largely due to small planting units (van Katwijk et al., 2016). However, observations from hundreds of restoration projects (van Katwijk et al., 2016) have led to major recent successes, such as the cost-effective restoration of 36 km^2^ of *Zostera marina* meadows in Virginia’s coastal waters, with major carbon removal benefits (Orth et al., 2020), as well as the long-term success of *Posidonia australis* restoration in SW Australia, again coupled with important carbon removal benefits (Marbà et al., 2015). Hence, seagrass restoration has a significant scope to contribute to climate action (Macreadie et al., 2021). There is ample scope for plant science to contribute to enhancing the success of seagrass restoration, through, for instance, the use of probiotic applications (Peixoto et al., 2022) or selective breeding of seagrasses used for restoration to enhance their resistance, and thereby restoration success, in areas experiencing marine heat waves (Zabin et al., 2022).

Lack of oxygen in seagrass soils, where oxygen penetration is limited to the top few mm of seagrass soils, slows down microbial degradation and the bioturbation activity of benthic fauna, thereby improving the efficiency of carbon burial. Anoxic sediments support sulfate-reducing bacteria, producing sulfide that is toxic to seagrass. However, seagrasses protect themselves from toxic sulfide intrusions by releasing oxygen through their roots, transported from photosynthetically produced oxygen in their leaves to their roots and rhizomes (Borum et al., 2006), thereby maintaining a protective oxidized layer a few millimeters thick around their roots and rhizomes (Brodersen et al., 2015). Oxygen transport from photosynthetic production sites to roots is enabled by the development of a lacunae system that provides gaseous connectivity between leaves, rhizomes, and roots (Borum et al., 2006). While continuous within organs, they are interrupted between organs by diaphragms one cell thick, perforated by interstitial pores (0.5–1.0 μm), which provide protection from flooding while allowing gas flow (Roberts et al., 1984). In addition, the below-ground tissues of seagrasses exhibit physiological adaptations which allow them to rely temporarily on anaerobic fermentative metabolism (Borum et al., 2006).

#### Known unknowns

Carbon concentrating mechanisms that allow seagrasses to support their high photosynthetic rates and circumvent boundary-layer rate-limiting effects are not fully resolved (Larkum et al., 2006). Seagrass carbon metabolism remains poorly understood and seems to neither fully conform to C_4_ nor Crassulacean acid metabolism (Larkum et al., 2006). Genomic analyses conducted to date have focused on the seagrass genome and ignored the rich community of endophytes. There is a growing number of analyses of the seagrass microbiome, including bacteria and fungi (Tarquinio et al., 2019; Garcias-Bonet et al., 2021; Torta et al., 2022), but they remain mostly descriptive and functional analyses are limited, despite evidence that endophytes may play a major role in supporting nutrient metabolism (Mohr et al., 2021) and detoxification (Crump et al., 2018). For instance, recently discovered cable bacteria in seagrass roots could alleviate critical sulfide toxicity and promote nutrient uptake by mobilizing soil iron and phosphorous with acidification associated with electrogenic sulfide oxidation, and by stimulating dissimilatory nitrate reduction to ammonium and even fixing N_2_ (Scholz et al., 2021).

#### Opportunities around unknown unknowns

Overall, limited progress has been made in applying modern concepts and tools of plant science to further our understanding of seagrass carbon removal, where an ecological focus prevails. This is not surprising given that seagrasses represent only 0.02% of angiosperm species and have little scope to emerge as model organisms. Yet, the strong selection pressure required for angiosperms to cope with life in the marine environment and anoxic, sulfide-rich sediments is likely to have generated novel mechanisms that can open new pathways in biotechnology. Understanding the carbon concentration mechanism of seagrass can open the door for hybrid photosynthesis technologies for carbon removal (Kornienko et al., 2018), while resolving the functional role of their microbiome can help improve the outcomes of seagrass restoration. The limited effort of plant science on seagrass research to date suggests the existence of “unknown unknowns” and, therefore, a potential for new discoveries that can lead to applications in carbon removal, conservation ecology and, more broadly, plant science.

## Can we improve photosynthesis?

### Photosynthesis: A key target for improving crop productivity, sustainability, and resilience in the face of climate change

#### (By Elizabeth A. Ainsworth and Andrew D.B. Leakey)

Photosynthesis heavily influences crop productivity, resource use efficiency, and sensitivity to stresses. Therefore, strategic engineering of photosynthetic metabolism and the morphological features of leaves that control carbon and water fluxes can: (1) increase the food, fuel, fiber, and feed produced by crops; while (2) reducing demand for water and improving agricultural GHG balance; and (3) making crops more resilient to future climatic and atmospheric conditions. Detailed models of photosynthetic metabolism (Zhu et al., 2012; Bellasio, 2019) and crop function can identify engineering strategies (Kromdijk et al., 2016; Leakey et al., 2019; Wu et al., 2019). Synthetic biology is also opening doors for novel photosynthetic systems to be custom designed to new environments (Zhu et al., 2020, and discussed below by Lu and Liao). Here, we discuss engineering for greater photosynthesis under near-future elevated atmospheric CO_2_ concentrations and temperatures, plus improved photosynthetic water use efficiency (WUE) and NUE.

Despite a general effect of higher atmospheric CO_2_ enhancing photosynthesis in C_3_ plants, global warming is expected to have profoundly negative consequences for crop photosynthesis and productivity by the middle to end of this century (Slattery and Ort, 2019). Rising temperatures also increase vapor pressure deficit (Ficklin and Novick, 2017), which may increase irrigation demand in the future and limit the potential yield of current crop genotypes grown under standard management practices (Ort and Long, 2014; DeLucia et al., 2019). Photosynthesis is a temperature-dependent process, with rates increasing to an optimum, then decreasing once that temperature optimum is exceeded (Moore et al., 2021). This temperature dependency reflects the biochemical processes that determine rate limitations, namely Rubisco activity (and the balance between photosynthetic carbon assimilation and photorespiration) and ribulose-1,5-bisphosphate regeneration. While in vitro Rubisco carboxylation rates increase beyond ∼50°C, decreased discrimination by Rubisco for oxygen and increased solubility of oxygen relative to CO_2_ with rising temperatures inhibit net photosynthetic carbon assimilation in temperate C_3_ crops at temperatures exceeding ∼30°C, due to increased photorespiration (Moore et al., 2021). Rubisco activase is a key target for improving photosynthesis at elevated temperatures because of the thermolability of the enzyme (Salvucchi and Crafts-Brandner, 2004) and the observation that activases from species or genotypes adapted to warmer climates are more thermostable (Scafaro et al., 2016).

Work in *Arabidopsis thaliana* suggested that simply overexpressing a thermostable Rubisco activase could improve photosynthesis and growth in high temperature conditions (Kurek et al., 2007), but that result was not translated to crops where overexpression of Rubisco activase resulted in lower Rubisco content (Fukayama et al., 2012, 2018). Studies in rice discovered that over-expression of both Rubisco and Rubisco activase were required for enhanced photosynthesis at both optimal and high temperatures (Qu et al., 2021; Suganami et al., 2021). A highly thermostable Rubisco activase identified in the Crassulacean acid metabolism plant *Agave tequilana* (Shivhare and Mueller-Cajar, 2017) and greater understanding of the mechanisms of thermostability in different Rubisco activase isoforms (Scafaro et al., 2019; Degen et al., 2020) provide potential guides for further improving thermotolerance in crops.

Another target for improving photosynthesis at elevated temperatures is reducing photorespiration, the process that recycles 2P-glycolate at the expense of ATP and NADH (Walker et al., 2016). A number of genetic engineering strategies have successfully demonstrated that photorespiration can be partially bypassed, resulting in improved photosynthetic carbon assimilation (Kebeish et al., 2007; Carvalho et al., 2012; South et al., 2019). Recently, transgenic tobacco (*Nicotiana tabacum*) was developed to recycle 2P-glycolate in the chloroplast via overexpression of plant malate synthase and Chlamydomonas (*C. reinhardtii*) glycolate dehydrogenase and simultaneous RNAi to downregulate a glycolate–glycerate transporter (South et al., 2019). When these plants were grown in the field at elevated temperatures (+ 5°C), they showed greater resilience to heat stress compared to wild-type (Cavanagh et al., 2022), providing strong proof-of-concept for this strategy.

Growth at elevated CO_2_ (550–600 ppb, which is in the range of predicted average atmospheric CO_2_ concentrations by 2050) generally enhances yields of C_3_ crops in major temperate growing regions (Ainsworth and Long, 2021). This primarily results from enhanced photosynthetic CO_2_ fixation driven by greater Rubisco carboxylation rates combined with inhibition of Rubisco oxygenation rates (Stitt, 1991). Even if C_3_ plants acclimate to elevated CO_2_ in the long term by downregulating investment in Rubisco content and electron transport capacity, photosynthesis is generally stimulated along with NUE (Leakey et al., 2009). Field experiments with transgenic plants overexpressing Calvin–Benson–Bassham (CBB) cycle enzymes further enhanced the benefits of elevated CO_2_ on carbon gain and yield by increasing photosynthetic electron transport capacity (Rosenthal et al., 2011; Köhler et al., 2017). If coupled with breeding or engineering to maintain high sink capacity, which is a prerequisite to maximizing the potential of photosynthetic enhancements in elevated CO_2_ (Ainsworth and Long, 2021), this provides a widely applicable pathway to a greater CO_2_-fertilization effect on yield.

Greater atmospheric CO_2_ also causes stomatal closure, resulting in lower transpiration and greater WUE (Leakey et al., 2009, 2019). This can reduce drought-induced stress and yield loss (Fitzgerald et al., 2016). However, interactions with abscisic acid signaling, canopy micrometeorology, and N fixation can also cause the CO_2_-fertilization effect on yield to be lost under hot and dry conditions (Gray et al., 2016). There is also significant uncertainty about which of these responses will occur in tropical locations where water availability, high temperatures, and soil fertility might be most limiting (Leakey et al., 2012). A possible target to improve yield in times and places of drought is to reduce the amount of water lost through stomata to the atmosphere relative to photosynthetic CO_2_ uptake, that is increasing WUE by reducing stomatal density or accelerating stomatal closing speed (Leakey et al., 2019). Modeling suggests that prioritizing reductions in water use over increases in carbon gain when trying to enhance WUE may lead to better yield outcomes in many growing environments for both C_3_ and C_4_ species, especially as atmospheric CO_2_ concentrations continue to rise (Leakey et al., 2019; Wu et al., 2019). Successful pursuit of this strategy would increase productivity while making currently marginal land viable for production, reduce freshwater use for irrigation, and make crops more resilient to climate change.

Crop productivity today is highly dependent on fertilizer application, which has negative environmental effects through nitrate run-off and release of the potent GHG nitrous oxide. The need for N inputs is strongly linked to the high N cost of photosynthetic proteins. However, there may be potential to re-invest N in different photosynthetic components to increase carbon gain and improve NUE (Evans and Clarke, 2019).

### Enhancing plant CO_2_ fixation through synthetic biology

#### (By Kuan-Jen Lu and James C. Liao)

**Figure 4:**
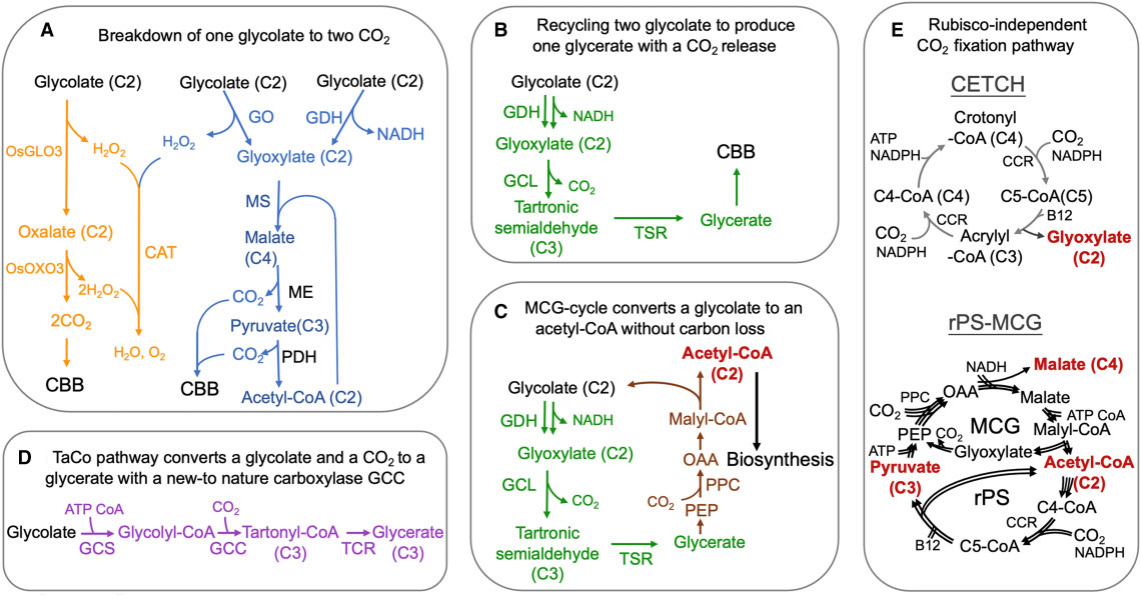
Synthetic biology approaches for recycling photorespiration and CO_2_-fixation pathways. A and B, Photorespiration engineered for breakdown of one glycolate to two CO_2_ molecules (A) or conversion of two glycolate to one glycerate plus one CO_2_ molecule (B). The CO_2_ released in the chloroplast is recycled back to the CBB cycle for carbon reassimilation (Maier et al., 2012; Shen et al., 2019; South et al., 2019). C, The MCG-cycle engineered to convert glycolate to acetyl-CoA without carbon loss (Yu et al., 2018b). D, Creation of a new enzyme, such as glycolyl-CoA carboxylase, to achieve glycolate recycling to produce glycerate with input of ATP and an additional CO2 molecule (Scheffen et al., 2021). E, Rubisco-independent CETCH and rPS-MCG synthetic CO_2_-fixation pathways (Luo et al., 2022).

Synthetic biology encompasses engineering natural or non-natural enzymes or pathways into plants to accomplish a designated purpose. In addition to the approaches discussed above, here we discuss attempts using synthetic biology to enhance CO_2_ fixation, focusing on recycling photorespiration products and CO_2_-fixation pathways (Figure 4).

#### Recycling photorespiration products

Plant photorespiration produces a nonproductive product, 2P-glycolate, through the oxygenase activity of Rubisco. 2P-glycolate is converted to glycerate in peroxisomes and to CO_2_ in mitochondria in a process requiring ATP and NADPH with CO_2_ and ammonium released (Walker et al., 2016). Current synthetic pathways for reducing photorespiratory CO_2_ loss involve the following types:

##### Breakdown of one glycolate (a C2 compound) to two CO_2_ in chloroplasts without ATP or NADPH consumption (Figure 4A)

The released CO_2_ can be reassimilated by Rubisco, and no ammonium would be released. For example, an engineered “GOC” pathway in rice consists of a glycolate oxidase (OsGLO3), an oxalate oxidase (OsOXO3), and a catalase (OsCATC) overexpressed in rice chloroplasts (Shen et al., 2019). Glycolate is converted to oxalate, which is completely oxidized to two CO_2_ by OsOXO3. OsCATC is required for decomposing H_2_O_2_, preventing plants from oxidative stress. Rice plants engineered with the GOC pathway showed a 22% increase in photosynthesis, but increases in yield were inconsistent and dependent on the season in field tests (Shen et al., 2019). An earlier example overexpressed a malate synthase (MS) from pumpkin (*Cucurbita pepo*), a catalase (CAT) from *Escherichia coli*, and a peroxisomal glycolate oxidase (GO) in Arabidopsis chloroplasts (Maier et al., 2012). In this manner, glycolate is completely oxidized to CO_2_ via both the heterologous and endogenous enzymes. The transgenic Arabidopsis had a greater rosette number and size with higher biomass under the ambient CO_2_, short-day conditions. However, introduction of the above three genes in tobacco did not result in increased biomass in greenhouse studies (South et al., 2019). When GO was replaced with Chlamydomonas glycolate dehydrogenase (GDH), which produces NADH instead of H_2_O_2_, transgenic tobacco showed higher carbon assimilation rates, resistance to photorespiration stress, and a significant increase in biomass in the field tests (South et al., 2019). Under high temperatures (+5°C), this pathway decreased yield loss by 11%–21% (Cavanagh et al., 2022).

##### Conversion of two glycolate (C2) to one glycerate (C3) with CO_2_ release in chloroplasts (Figure 4B)

The synthetic pathway originated from *E. coli*, consisting of dehydrogenase (GDH), glyoxylate carboligase (GCL), and tartronic semialdehyde reductase (TSR). Unlike the first approach, this synthetic pathway preserves 75% of carbon from two glyoxylate to produce one glycerate, which is returned to the CBB cycle (Kebeish et al., 2007). The remaining carbon is CO_2_ produced via GCL. Expressing the above genes in Arabidopsis chloroplasts increased the growth rate and biomass yield. This synthetic pathway was shown to benefit crop plants such as *Camelina sativa* and potato (*Solanum tuberosum*) in greenhouse and growth chamber conditions (Nolke et al., 2014; Dalal et al., 2015).

##### Fixation of an additional CO_2_ to compensate for the carbon loss by GCL (Figure 4C)

The synthetic malyl-CoA glycerate (MCG) cycle also uses GCL, and TSR to convert two glyoxylates to glycerate, which is then converted to phosphoenolphyruvate (PEP). The oxygen-insensitive PEP carboxylase (PPC) then carboxylates CO_2_ and PEP to OAA (C4), followed by splitting OAA to acetyl-CoA and glyoxylate (Yu et al., 2018b). The glyoxylate is then recycled in the GCL reaction. The net result is the conversion of glyoxylate (or glycolate) to a productive biosynthetic product, acetyl-CoA, without carbon loss. The MCG cycle has been accomplished in *Synechococcus elongatus PCC7942*, a photoautotrophic cyanobacterium (Yu et al., 2018b). Compared to the wild-type, the strain expressing the MCG cycle fixed higher amounts of CO_2_ to produce more acetyl-CoA and its derived compound ketoisocaproate, an intermediate in leucine biosynthesis.

##### Fixation of an additional CO_2_ to glycolate after activation (Figure 4D)

An elegant tartronyl-CoA (TaCo) pathway was demonstrated recently, in which glycolate is activated to glycoly-CoA, which is then caboxylated to tartronyl-CoA and then to glycerate. This approach requires a new-to-nature enzyme, glycolyl-CoA carboxylase, which was developed by rational design and high-throughput screening (Scheffen et al., 2021).

#### Rubisco-independent, synthetic CO_2_ fixation pathways

Six Rubisco-independent CO_2_-fixation pathways in microorganisms have been identified in nature (Berg, 2011), and a number of theoretical synthetic pathways have been designed in silico based on reported enzyme activities and thermodynamics (Bar-Even, 2018). The first step in implementing synthetic pathways is to demonstrate the pathway feasibility in a cell-free system. This in vitro demonstration requires in-depth processes in solving problems in co-factor regeneration, enzyme stability, and pathway control. Through these processes, incompatibility of enzyme reactions, kinetic barrier, and thermodynamic limitations can be identified. To date, two Rubisco-independent synthetic CO_2_-fixing pathways, CETCH and reductive pyruvate synthesis (rPS)–MCG (Figure 4E), have been demonstrated, and achieved similar or increased CO_2_ fixation rates in vitro compared with the CBB cycle in vivo (Schwander et al., 2016; Luo et al., 2022). The CETCH pathway consists of 17 enzymes from different organisms (Schwander et al., 2016). An oxygen-insensitive carboxylase/reductase (CCR) from *Methylorubrum extorqens* was chosen as the carboxylase to fix CO_2_ in the CETCH cycle because of its high carboxylase activity and broad substrate range. The carboxylation substrate acrylyl-CoA and crotonyl-CoA in CETCH were regenerated to complete the cycle for continuous fixation of CO_2_. The fixed carbon is converted to glyoxylate as the output.

The rPS–MCG cycle consists of two parts (Luo et al., 2022). The first utilizes the MCG cycle described above. In the second part, rPS converts acetyl-CoA to pyruvate through a series of reactions that takes two acetyl-CoA to make a crotonyl-CoA, which is carboxylated by CCR to produce a C5 compound. The C5 compound is split into a C3 (pyruvate) and C2 (acetyl-CoA) through a series of carbon rearrangement reactions that complete the cycle. The rPS–MCG cycle exhibits a self-replenishing feature as it can export any of its intermediates as a product, such as acetyl-CoA (C2), pyruvate (C3), and malate (C4). This self-replenishing characteristic is also seen in almost all naturally evolved cycles. Since the output C2, C3, or C4 intermediates are essential for cell growth, it is potentially malleable for in vivo engineering. Introduction of the CETCH cycle or the rPS–MCG cycle in a plant would require the activity of many heterologous enzymes, along with co-enzyme B12, which is absent in plants. Hence, enzyme design, pathway evaluation in prokaryotes, plant-associated microbiome engineering, and various genome editing strategies have been proposed to facilitate this process (Erb et al., 2017; Gupta et al., 2021; Ke et al., 2021).

### Engineering carbon dioxide-responsive C_3_ crops to sustain higher productivity under a CO_2_-rich, warmer climate

#### (By Rajeev N. Bahuguna and S. V. Krishna Jagadish)

C_4_ plant species are overrepresented in agriculture systems and have substantially higher productivity compared to C_3_ crops mainly due to higher photosynthetic efficiency (Rao et al., 2012; Sales et al., 2021). Yet a number of C_3_ crops are important food sources for millions of people globally, including cereals such as wheat, rice, barley (*Hordeum vulgare*), oats (*Avena sativa*), and many vegetable and tree crops. Therefore, efforts to increase the photosynthetic efficiency and productivity of C_3_ crops are underway to help meet the increasing global food demand (Cui 2021). The high CO_2_ saturation point for photosynthesis of C_3_ plants (intercellular CO_2_ levels ∼600 µmol mol^−1^) makes them more responsive to elevated CO_2_ than C_4_ plants, which are saturated for CO_2_ under current atmospheric CO_2_ levels (Loladze, 2014; Dingkuhn et al., 2020; Kundu et al., 2022). Thus, C_3_ crops provide a unique opportunity to harvest more carbon from a CO_2_-rich environment and convert it to biomass and yield (Broberg et al., 2019; Ainsworth and Long, 2021).

**Figure 5:**
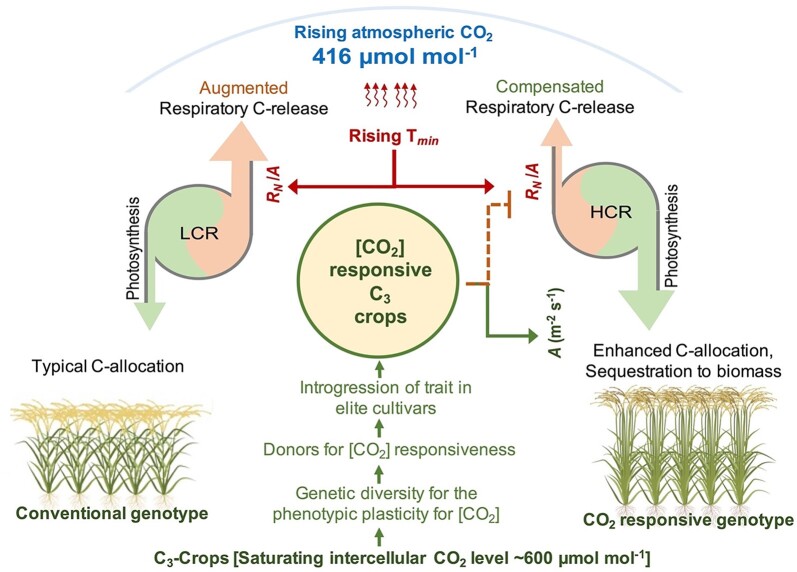
Schematic diagram showing average annual atmospheric [CO_2_] level for 2021 and the effect of rising night temperature (*T*_min_) on rice productivity by enhanced respiration: photosynthesis ratio (*R_N_/A*) resulting in augmented release of carbon at the cost of biomass and yield in conventional genotypes. On the contrary, introgression of CO_2_-responsiveness trait in C_3_ crops facilitates enhanced carbon sequestration and allocation of additional carbon into biomass, and compensating *T*_min_-induced carbon losses. LCR, least CO_2_-responsive; HCR, high CO_2_-responsive.

In contrast to the positive effect of CO_2_ on C_3_ photosynthesis, the global rise in temperature is a major factor limiting the yield of major cereal crops (Lobell and Gourdji, 2012; Teixeira et al., 2013; Zhao et al., 2017). A rise in night temperature has been shown to have a large impact on the productivity of C_3_ crops such as rice (Peng et al., 2004; Welch et al., 2010) and wheat (Hein et al., 2020, 2022; Impa et al., 2021). Recent studies suggest that high night temperature (HNT) is related to physiological changes such as an increased rate of night respiration (*R_N_*) and a reduced rate of starch accumulation in developing grains in rice (Bahuguna et al., 2017; Shi et al., 2017), wheat (Narayanan et al., 2016a, 2016b; Impa et al., 2020), and barley (García et al., 2015, 2016). Hence, the positive effect of CO_2_ on C_3_ photosynthesis and augmented rate of night respiration under HNT have opposing effects on carbon-balance dynamics under CO_2_-rich, warmer environments (Song et al., 2014; Dusenge et al., 2019). While the sensitivity of *R_N_* to a rise in temperature is well documented (Atkin and Tjoelker, 2003), variable effects of the long- and short-term impact of elevated CO_2_ on *R_N_* have been reported, ranging from direct inhibition of respiration to no significant impact or even an increase under long-term exposure to elevated CO_2_ (Griffin et al., 1996; Gonzalez-Meler et al., 1996, 2004; Ziska and Bunce, 1998; Drake et al., 1999; Baker et al., 2000; Davey et al., 2004; Ayub et al., 2014). However, none of these studies considered the genetic background for CO_2_ responsiveness, which could be a major determinant of the effect of elevated CO_2_ on *R_N_*, and carbon balance dynamics in C_3_ crops (Figure 5).

Despite the well-documented photosynthetic enhancement of C_3_ crops under elevated CO_2_ (Leakey et al., 2009), active selection in C_3_ crops for CO_2_ responsiveness has not been given adequate attention (Ziska et al., 2012; Dingkuhn et al., 2020). The complexity of field-based CO_2_ enrichment facilities and space constraints for screening and characterizing a large number of genotypes remain major bottlenecks for identifying potential CO_2_-responsive genotypes. Recently, Shimono et al. (2014) and Kikuchi et al. (2017) demonstrated that altering planting density provides a means of assessing phenotypic plasticity in rice genotypes under enhanced resource availability (e.g. space, light, nutrients). Interestingly, genotypes responsive to higher available resources under low planting density responded similarly under an elevated CO_2_ environment (Shimono et al., 2014). Subsequently, in a series of field experiments, Bahuguna et al. (2022) assessed the variable phenotypic plasticity of 194 diverse rice genotypes by measuring parameters related to photosynthesis, biomass, and yield under different planting densities. A wide genetic variability observed for the phenotypic plasticity under a resource-rich environment showed a strong relationship (*R*^2^ = 0.71) with CO_2_ responsiveness under realistic CO_2_ conditions using a field-based free air CO_2_ enrichment facility. Further, the high CO_2_-responsive (HCR) genotypes showed significantly higher rates of photosynthesis (*A*) and lower rates of *R_N_* resulting in a lower *R_N_/A* ratio as compared to the least CO_2_-responsive (LCR) genotypes. Interestingly, elevated CO_2_ was identified as the major driver influencing carbon-balance dynamics and the phenotypic response of HCR genotypes resulting in higher biomass and yield under elevated CO_2_ + HNT conditions, whereas the LCR genotype was severely affected by HNT despite exposure to elevated CO_2_.

This study demonstrated that the impact of HNT on grain yield, total biomass, and grain weight was compensated by elevated CO_2_, but this response was mainly confined to the HCR genotypes (Bahuguna et al., 2022). Thus, LCR or conventional genotypes are expected to lose biomass and yield under an elevated CO_2_, warmer climate due to augmented respiratory carbon losses, whereas HCR genotypes could accumulate more carbon per unit area and maintain their biomass and yield by compensating for carbon losses under HNT (Figure 5). In addition, the ability to fix additional carbon with a lower respiration-to-photosynthesis ratio in HCR genotypes provides an opportunity to sequester a substantial amount of carbon into biomass. There is, however, a need for prediction models for simulated carbon fluxes at temporal and spatial scales to assess the carbon sequestration potential of CO_2_-responsive C_3_ crops. In conclusion, the introgression of a ‘CO_2_-responsiveness’ trait into elite rice varieties and other C_3_ crops could help sustain and enhance crop yield in a warmer environment.

### The C_4_ rice project

#### (By Jane Langdale)

In the majority of photosynthetic organisms, both in water and on land, CO_2_ is fixed by Rubisco into the three-carbon compound 3-phospho-glycerate, the first intermediate of the CBB cycle. The efficiency of this C_3_ photosynthetic pathway is compromised because Rubisco also reacts with oxygen, forming 2-phospho-glycolate, which has to be detoxified in the energetically costly photorespiratory pathway (Walker et al., 2016). Because of this energetically wasteful competitive reaction, the decrease in atmospheric CO_2_ levels that occurred during the Oligocene (Pearson et al., 2009) would have been accompanied by photosynthetic inefficiencies at a global scale.

**Figure 6:**
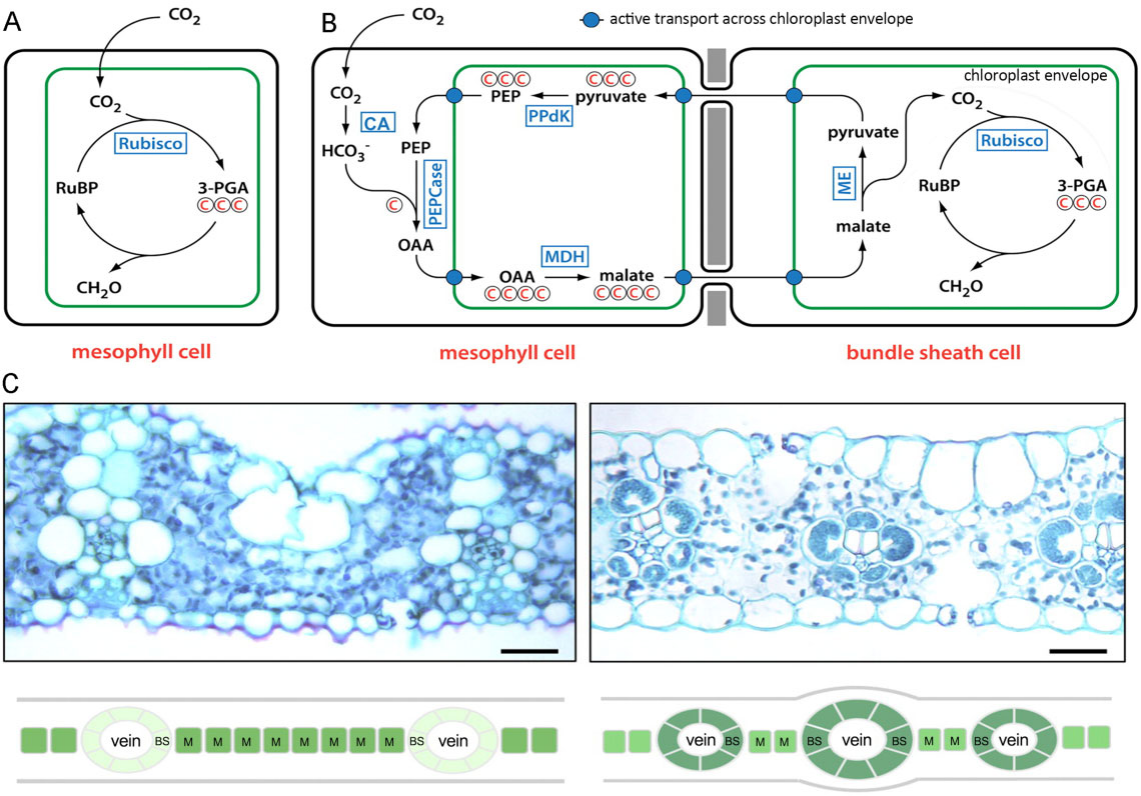
Schematics of C_3_ CBB and NADP-ME C_4_ Cycles. A, CBB C_3_ cycle. B, NADP-ME C_4_ cycle. C, Transverse leaf sections and corresponding schematics of C_3_ rice (left) and C_4_ maize (right). Bars = 30 μm. Adapted from Langdale (2011), Figures 1 and 3.

The reported drop from ∼800 to ∼400 ppm atmospheric CO_2_ during this period is thought to have driven, at least in part, the evolution of the C_4_ photosynthetic pathway that concentrates CO_2_ at the site of Rubisco and thus minimizes photorespiration (Sage, 2016). In the C_4_ pathway, CO_2_ is initially fixed by phosphoenolpyruvate carboxylase (PEPCase), which is oxygen insensitive. This carboxylation reaction occurs in the outer mesophyll cells of the leaf, with the four-carbon reaction product subsequently transported to inner bundle sheath cells for decarboxylation and re-fixation by Rubisco in the Calvin cycle (Figure 6). Given the specialized leaf anatomy and compartmentalization of metabolic reactions required for C_4_ function, evolution of the pathway must have involved functional modification of multiple genes, including those encoding enzymes, metabolite transporters, and regulators of cell-type patterning. Despite this apparent complexity, the C_4_ photosynthetic pathway evolved over 60 times independently and is represented in diverse families of flowering plants (Sage, 2016). The adaptive success of the C_4_ photosynthetic strategy is demonstrated by the fact that just 2% of plant species utilize the pathway but C_4_ plants are responsible for ∼25% of terrestrial primary productivity (Still et al., 2003).

#### Why C_4_ rice?

In addition to strategies that aim to improve the efficiency of the C_3_ photosynthetic pathway (discussed above, and see Ort et al., 2015; Johnson, 2022) or to introduce Crassulacean acid metabolism into C_3_ plants (Schiller and Bräutigam, 2021), the enhanced efficiency of C_4_ photosynthesis provides a potential engineering opportunity for improved yield and resilience against abiotic stresses in C_3_ crops. Although the C_4_ pathway utilizes two extra ATP molecules per CO_2_ fixed than the C_3_ pathway, in warm and dry environments where dissolved oxygen conditions are relatively high, these energy costs are offset by those not spent on photorespiration (3.5 ATP per O_2_ fixed). In general, C_4_ plants also use less water (Kocacinar et al., 2008) and N (Evans and von Caemmerer, 2000) per CO_2_ fixed and have substantially faster growth rates (Monteith 1978). Physiological models that incorporate these factors predict that if C_4_ traits could be introduced into C_3_ plants, enhanced radiation, N, and WUEs could generate substantial yield increases, particularly in warm environments where crops are rainfed and fertilizer applications are limited (Mitchell and Sheehy, 2006).

Importantly, the level of atmospheric CO_2_ at which C_4_ outcompetes C_3_ is dependent on temperature; C_4_ is favored below 550 ppm CO_2_ at 35°C, 450 ppm at 30°C, and 350 ppm at 25°C (Ehleringer et al., 1997). Although future predictions of atmospheric CO_2_ levels differ depending on fossil fuel usage scenarios, with current levels at 419 ppm and annual increases of 2–3 ppm over the last decade (https://gml.noaa.gov/ccgg/trends/gl_gr.html), the status quo would result in atmospheric CO_2_ levels of ∼500 ppm by 2050. C_4_ plants could thus outperform C_3_ plants where temperatures exceeded ∼33^o^C, which given climate warming predictions could be much of the global agricultural landscape for at least part of the year. Leaving predictions aside, long-term field experiments at elevated (+180 ppm) CO_2_ demonstrated that the biomass of C_3_ but not C_4_ grasses was enhanced over the first 12 years of the project but then C_4_ outperformed C_3_ in the following 8 years (Reich et al., 2018). This switch was correlated with net N mineralization rates in the soil, which were initially enhanced by elevated CO_2_ in C_3_ plots but were later depressed. Despite the difficulties in predicting exactly how plants will respond to global change, C_4_ engineering is thus a plausible strategy, albeit one with significant challenges.

#### Strategy

The C_3_ species rice is an obvious target for C_4_ engineering because it is one of the world’s top three staple crops and in many parts of Asia it is the major source of calorie intake. With predicted population increases, the one hectare of land that provided enough rice to feed 27 people in Asia in 2007 will need to support at least 43 people by 2050—a 60% increase in demand (Zeigler, 2007). Successful conversion of a C_3_ plant into one that utilizes the C_4_ pathway requires that leaf anatomy be modified to reduce the number of mesophyll cells between veins to the extent that there is an approximate 1:1 ratio of mesophyll:bundle sheath cells in the leaf; that chloroplast development is activated in the normally achlorophyllous bundle sheath cells; and that C_4_ pathway enzymes and metabolite transporters are compartmentalized and functional in either the mesophyll or bundle sheath cells.

When the C_4_ Rice Project (www.c4rice.com) was initiated, genes encoding all of the enzymes of the C_4_ pathway had been identified in maize and other C_4_ species, as had some of the genes encoding metabolite transporters (reviewed in Langdale, 2011), but regulators of C_4_ leaf anatomy had not been identified. The strategy to introduce C_4_ traits into rice was thus three-pronged: (1) introduce compartmentalized C_4_ metabolism into existing bundle sheath cells and the mesophyll cells immediately adjacent to them by expressing maize genes in specific cell-types of rice; (2) activate chloroplast development and photosynthesis in existing bundle sheath cells by expressing a known regulator of chloroplast development in maize (the *Golden2* [*ZmG2*] gene; Hall et al., 1998); and (3) identify regulators of C_4_ leaf anatomy in maize with a view to future manipulation in rice. The ultimate goal was to combine the metabolic prototypes generated in the first two strands with the anatomical prototype.

Much of the first decade of the project was spent developing tools in rice to enable this strategy, for example robust transformation pipelines, cell-type-specific promoters, and modular cloning technology. Ongoing research continues to characterize potential regulators of C_4_ leaf anatomy and to evaluate whether manipulation in rice can modify cell-type patterning in the leaf (Wang et al., 2013a, 2017a; Schuler et al., 2018; Hughes et al., 2019; Hughes and Langdale, 2020, 2022)—but much more discovery research is needed before an anatomical prototype can be designed and engineered (Sedelnikova et al., 2018). Recent work has, however, made progress toward engineering C_4_ metabolic prototypes.

#### Progress

Maize genes encoding C_4_ pathway enzymes have been expressed in specific cell types of both an elite cultivar of *indica* rice (IR64) and a model cultivar of *japonica* rice (Kitaake), and in both cases the pathway is partly functional. Specifically, primary carboxylation by PEPCase is seen in mesophyll cells, but subsequent decarboxylation in bundle sheath cells has yet to be detected (Lin et al., 2020; Ermakova et al., 2021). Creating a fully functional cycle will require a better understanding of metabolite flux within and between the two cell types, which may require the development of more sensitive detection methods. In a second advance, chloroplast development has been activated in the normally achlorophyllous bundle sheath cells of rice, through constitutive expression of *ZmG2* (Wang et al., 2017b). No fitness penalty was observed in greenhouse-grown lines expressing *ZmG2*, in either IR64 or Kitaake backgrounds (Wang et al., 2017b) and although only evaluated in the nonelite Kitaake background, field-grown lines overexpressing *ZmG2* exhibited up to 30% yield increases (Li et al., 2020). These examples validate the overall engineering strategy but there is still a long way to go before a full transition to C_4_ can be achieved in any C_3_ species.

## Can we develop climate-resilient crops?

### The trait development pipeline: Bridging the gap between upstream science and breeding for adaptation to climate change

#### (By J. Damien Platten and Amelia Henry)

**Figure 7:**
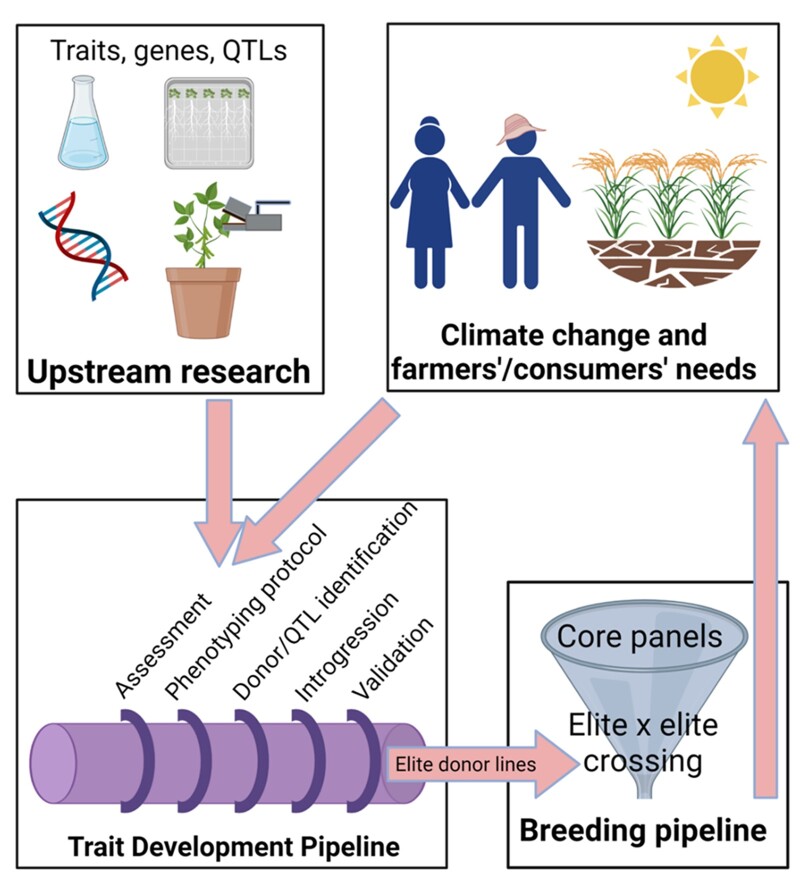
The Trait Development Pipeline for delivery of valuable stress tolerance traits/genes/QTLs from upstream research into the elite breeding pool for improvement of crop productivity under climate change. The Trait Development Pipeline is organized into six stages: 1) guidelines for prioritizing traits (assessment), 2) defining standards for phenotyping protocols, 3) identifying donors and QTL (including refining marker quality metrics), 4) introgressing and 5) validating traits/genes/QTLs into elite genetic backgrounds to develop the elite donor lines that are 6) handed to the breeding program for crossing. Those elite donor lines will then be systematically crossed and tested in target environments where climate change is increasingly affecting the degree of abiotic stress affecting crop production. Created with BioRender.com

Improving the adaptability of crops is a key strategy to mitigate the effects of climate change on productivity (Aggarwal et al., 2019). We focus on rice breeding in this section; however, the pipeline we describe (Figure 7) could easily be extended to other crops, taking into consideration the challenges and parameters unique to each species. For example, the platform is being adopted across the CGIAR partnership for global food security (https://www.cgiar.org/) for other mandate crops. In rice breeding, abiotic stress tolerance was not a selection target during the Green Revolution, and some evidence suggests that stress tolerance was even selected against due to tight linkage between stress tolerance loci and loci conferring unfavorable agronomic traits (Vikram et al., 2015). Subsequently, a range of breeding approaches has been taken to improve stress tolerance, including introgression of quantitative trait loci (QTLs) for stress tolerance traits as well as direct selection for grain yield under stress using traditional varieties as the sources of stress tolerance. Characterization of stress-tolerant varieties has revealed that combinations of physiological traits have been affected by selection for yield under stress (Anantha et al., 2016; Kumar et al., 2021), which may explain some of the difficulty in developing superior varieties through introgression of single genes/QTLs. With a few exceptions (i.e. *Sub1* varieties for submergence such as Swarna-sub1 [Mackill et al., 2012] and drought-tolerant DRR dhan 42 [IR64 *qDTY_2.2_* + *qDTY_4.1_*; Swamy et al. 2013]) the majority of recently released stress-tolerant varieties were conventionally bred (i.e. by crossing and selecting over several generations).

The use of genes and QTLs through marker-assisted selection could shorten the breeding process. Although hundreds of stress-tolerance genes, QTLs, and physiological mechanisms have been identified, only a small number of these research outputs have been used in breeding (Wissuwa et al., 2016; Cobb et al., 2019; Platten et al., 2019) and the frequency of known abiotic stress QTLs in the current elite breeding material remains low (Juma et al., 2021). There is thus a need to bridge the gap between upstream science and breeding for adaptation to climate change so that valuable traits/genes/QTLs are more actively utilized in breeding pipelines.

Modern breeding strategies have shifted to a paradigm of population improvement based on elite x elite crossing (Juma et al., 2021) within core panels, which for stress-prone areas have been selected from the most stress-tolerant genotypes available (i.e. Khanna et al., 2022). This strategy presents significant opportunities for upstream plant biologists to contribute to breeding efforts. With a defined list of genetic backgrounds (many of which have already been sequenced; see, for example, Mansueto et al., 2017) to which potential stress tolerance traits/genes/QTLs can be compared, those that best complement the elite breeding pool can be prioritized. However, although traditional varieties are the most promising source of stress tolerance, they also typically possess detrimental traits that make them unsuitable for use in elite × elite crossing. A defined protocol is needed to deliver useful traits/genes/QTLs from traditional varieties into elite backgrounds and into the breeding pool.

In seeking to bridge the gap between upstream plant science and breeding for stress tolerance, an understanding of breeding program needs is critical. In the briefest terms, reliability is key: a gene/QTL must reliably improve the target trait, in relevant elite genomic backgrounds and in relevant environments (field locations). Therefore, the growth stages, genetic backgrounds, and environmental conditions relevant to breeding programs should be reflected in the study systems used in upstream research. One example is in the validation of candidate genes for stress tolerance: this is frequently done in the background of *japonica* rice due to the established transformation protocols. However, the rice type preferred in most stress-prone rice-growing regions is *indica*, which grows better than temperate *japonica* rice in field trials in the tropics. Use of the relevant genetic background is important because stress tolerance alleles are often absent in *japonica* genomes and thus their level of stress tolerance is more easily improved, exaggerating apparent effect size. Other recommendations for increasing the likelihood of upstream research outputs being taken up by breeding are to take additional steps such as validation of identified QTLs in relevant elite genomic backgrounds and to link with researchers who can evaluate the material under field conditions.

On the other side of the gap, downstream science must make released varieties and advanced breeding lines more accessible to upstream scientists. Familiarity with this material is important because in some cases, key genes have been identified and advocated as promising breeding targets without the recognition that they are already present in the breeding pool. Furthermore, the availability of improved material to upstream researchers will help to ensure that target loci are effective in those genetic backgrounds. Downstream science also must stay up to date and gain access to the most recently identified traits/genes/QTLs to incorporate into the breeding program, while at the same time effectively connecting with local breeders who can conduct widespread testing and who understand the needs of farmers in stress-prone regions. This “bridge building” among scientific disciplines is critical to the development of more efficient pipelines that bring novel improvements to crops for climate change-affected farmers.

To strengthen linkages between upstream and downstream development efforts, a framework has been developed that organizes and codifies trait development efforts. This “Trait Development Pipeline” developed at the International Rice Research Institute (Figure 7) applies stage gate systems widely used in industry (Covarrubias-Pazaran et al., 2022) to assess trait development progress against defined advancement criteria. The organization by stages enables external review of the progress at each stage and provides decision points on whether to proceed, giving an opportunity to discontinue efforts that are not likely to make an impact in breeding programs.

The current Trait Development Pipeline is organized into six stages that link a variety of research disciplines, and the pipeline provides a structure that gives a framework for teamwork between these areas. The pipeline starts with an assessment of the trait of interest in the context of priority traits needed by farmers and consumers that are not already present in the elite breeding pool (see “Product concepts” and “Market segments”; Covarrubias-Pazaran et al., 2022). A set of criteria regarding the availability and reliability of phenotyping protocols for the trait, potential donor genotypes, mapping populations, QTLs, and markers determine advancement to subsequent stages in the Trait Development Pipeline. The pipeline is dynamic and subject to modification over time based on researcher feedback and as techniques and technologies change. The outputs of the Trait Development Pipeline are validated donor lines containing new traits/genes/QTLs in a fully elite background. These “elite donor lines” can be used in the elite × elite crossing work to improve the most advanced breeding lines which will be evaluated in multilocation trials, evaluated by local researchers, and considered for release as varieties for dissemination to farmers. In this way, the outputs of trait/gene/QTL discovery realize an ongoing impact across the breeding pool rather than improving just a single variety. Such sustained improvement through mainstream breeding programs will facilitate the deployment of new technologies to as many climate-change-affected crop production market segments as possible.

Application of the pipeline to known genes and QTLs helps to identify gaps in knowledge and products available, and addressing these gaps is already enabling the rapid introduction of a wide variety of genes contributing to disease resistance, heat, drought, cold, and salinity tolerance into mainstream rice breeding efforts. Breeding programs are thus able to respond in a far more agile manner to changing climate and market demands. As these genes are deployed into elite backgrounds, it becomes easier for small breeding programs to also leverage their value; the “heavy lifting” of eliminating highly unfavorable genomic backgrounds, breaking linkage drag, and developing coupling-phase linkages has been done, so only minimal or no additional effort is required to move the new genes to other elite breeding programs. Thus, the value of new genes is no longer exclusively available to large, well-resourced programs.

### Enhancing climate resilience through the use of crop wild relatives

#### (By Damaris A. Odeny)

Crop wild relatives (CWRs) are wild species that are closely related to domesticated crops and can be used for crop improvement. Breeders have traditionally used CWRs as sources of superior traits, including key traits for enhancing adaptation to climate change (Dempewolf et al. 2014). The main breeding objectives for climate change adaptation include resilience to abiotic stresses (drought, heat, salinity, and flooding/waterlogging) and biotic stresses brought about as a result of the increase in atmospheric CO_2_ and elevated average temperatures. Here, we provide examples of recent progress in the use of CWRs in managing these stresses and highlight specific areas where work is needed. Table 1 provides a summary of the use of CWRs in breeding for tolerance to abiotic stresses.

**Table 1.** Examples of wild relatives used to enhance abiotic stress tolerance in cultivated crops

Crop	More resilient wild species	Trait of interest	Reference
Wheat	*Aegilops cylindrica*	Drought	Pour-Aboughadareh et al. (2017)
	*Ae. crassa*		
	*Ae. caudata*		
	*Triticum urartu*		
	*T. monococcum*	Heat	Khanna-Chopra and Viswanathan (1999); Peng et al. (2013); El Haddad et al. (2021)
	*T. dicoccoides*		
	*Ae. speltoides* ssp. *liqustica*		
	*T. ararticum*		
	*Ae. speltoides*	Salinity	Ahmadi et al. (2018)
	*Ae. caudata*		
	*Ae. cylindrica*		
	*T. boeoticum*		
Sorghum	*Sorghum macrospermum*	Drought	Cowan et al. (2020)
	*S. brachypodum*		Ochieng et al. (2020)
	*S. arundinaceum*		
	*S. sudanense*		
	*S. purpureosericeum*		
Banana	*Musa balbisiana;*	Drought	Eyland et al. (2022)
	*M. acuminata* ssp. *errans*		
Rice	*Oryza rufipogon*	Salinity	Tin et al. (2021)
	*O. nivara; O. coarctata*		Zhang and Xie (2014)
	*O. nivara; O. rufipogon*	Flooding	Niroula et al. (2012)
	*O. meridionalis* Ng.	Heat	Scafaro et al. (2012); Scafaro et al. (2016); Scafaro et al. (2018)
	*O. australiensis*		
Maize	*Zea nicaraguensis*	Flooding	Mano et al. (2006);
	*Z. luxurians*		Mano et al. (2005);
	*Z. mays* ssp. *huehuetenangensis*		
	*Z. diploperennis*	Drought	Shaibu et al. (2021)
Tomato	*Solanum cheesmaniae;*	Salinity	Dehan and Tal (1978); Shalata and Tal (1998); Mittova et al. (2002); Frary et al. (2010); Pailles et al. (2020)
	*S. pennellii*		
	*S. galapagense*		
	*S. pimpinellifolium*	Heat	Driedonks (2018)
Tepary bean	Wild *Phaseolus acutifolius*	Drought	Buitrago-Bitar et al. (2021)
Adzuki bean	*Vigna nakashimae; V. riukiensis*	Salinity	Yoshida et al. (2016)
Eggplant	*Solanum insanum*	Salinity	Brenes et al. 2020)
Chickpea	*Cicer reticulatum*	Drought	Moenga et al. (2020)
Sugarcane	*Saccharum spontaneum*	Salinity	Kasirajan et al. (2021)

#### Drought stress

Despite the complexity of drought stress (Ilyas et al., 2021), CWRs have been reported that are more efficient than crop relatives in drought-related physiological processes such as higher WUE, higher CO_2_ assimilation, deeper root systems, more efficient regulatory networks, leaf curling, and stomatal closure, as well as showing an abundance of allelic diversity within candidate genes. For example, higher WUE, higher carbon assimilation, and greater carboxylation efficiency were reported in wild lettuce (*Lactuca serriola*; Eriksen et al., 2020), and Moenga et al. (2020) reported novel divergent drought tolerance mechanisms in wild chickpea (*Cicer reticulatum*) that would be a great resource for improving cultivated chickpea (*Cicer arietinum*). Drought-related transcription factors of the *Asr* (abscisic acid, stress, ripening) family have a high level of diversity in CWRs (Cortés et al. 2012) that might be further exploited to improve cultivated crops. Most drought studies to date in CWRs have focused on major crops, and there is tremendous scope to undertake similar studies in minor and under-researched crops.

#### Heat stress

Heat stress is one of the greatest concerns for crop production considering the increasing effects of climate change. The wild wheat relatives *Triticum monococcum*, *T. dicoccoides*, and *Aegilops speltoides* ssp. *liqustica* and CWR-derived wheat genotypes were among the most heat tolerant when tested alongside elite wheat genotypes (Peng et al., 2013; El Haddad et al., 2021). Similar observations have been made in wild rice, *Oryza meridionalis* Ng. and *O. australiensis*, in which heat tolerance was associated with a more stable activation of Rubisco (Scafaro et al., 2016). Overexpressing a thermostable variant of Rubisco activase from CWR significantly improved yield in domesticated rice (*Oryza sativa* L.; Scafaro et al., 2018). More studies on the physiological and molecular basis of heat tolerance in wild versus domesticated species are needed to enhance the deployment of novel heat tolerant alleles in crop improvement.

#### Salinity tolerance

Halophytic plants adapt to salinity through three distinct mechanisms, all of which have been identified in various CWRs: osmotic stress tolerance, Na^+^ or Cl^−^ exclusion, and tolerance of tissue to accumulated Na^+^ or Cl^−^. Wild relatives of adzuki bean, *Vigna nakashimae* and *V. riukiensis*, prevented Na^+^ accumulation in roots and stems, and tolerated accumulated Na^+^, respectively (Yoshida et al., 2016). A wild relative of tomato, *Solanum pennellii*, showed greater induction of antioxidant activity than cultivated tomato (*Solanum lycopersicum* L.) under salt stress (Frary et al., 2010). Salinity tolerance has also been reported in *Oryza glaberrima* (Platten et al., 2013), *Hordeum spontaneum* (Kiani-Pouya et al., 2020), and in *Aegilops* spp. (Zamani Babgohari et al., 2013).

#### Flooding tolerance

Flooding tolerance has been mainly studied in rice leading to the identification of the *SUB1* locus (Mackill et al., 2012). Additional submergence-tolerant alleles (*SUB1A-1*) were identified from wild rice species *O. nivara* and *O. rufipogon*, together with a likely presence of other submergence mechanisms in other wild rice accessions (Niroula et al., 2012). Two anaerobic germination QTLs (*qAGP1* and *qAGP3*) from *O. nivara* introgression lines (Liu et al., 2021a) potentially can be used to enhance flooding tolerance in elite *SUB1* genotypes, which are not always tolerant to anaerobic conditions during germination. Wild species with tolerance to waterlogging/stagnant flooding have been reported to possess unique alleles for aerenchyma formation (Zhang et al., 2017), or to provide a stronger barrier to radial oxygen loss (Pedersen et al., 2021). The availability of these different sources of flooding/waterlogging resistance in CWRs provides an opportunity to introgress the beneficial alleles into elite varieties, especially where genomics-assisted introgression and selection is possible.

#### CWRs as sources of resistance/tolerance to biotic stress

Introgression of disease resistance genes into cultivated crop species is perhaps the most beneficial use of CWRs in crop improvement to date. Major genes have been introgressed from CWRs for resistance to late blight (*Phytophthora infestans*) in potato (*Solanum tuberosum* L.; Ghislain et al., 2019), blast disease (*Magnaporthe oryzae*) resistance in rice (Yoshida and Miyashita, 2009) and several other key pathogens in wheat (Rani et al., 2020) and tomato (Sharlach et al., 2013), just to mention a few. Pest resistance also benefited from CWRs (Therezan et al., 2021), among the most recent being the introduction of fall armyworm (*Spodoptera frugiperda*) resistance from wild relatives of maize (Singh et al., 2022). Climate change-related warmer average temperatures and altered weather patterns are contributing to altered patterns in the occurrence of crop pests and pathogens and the emergence of new pests and pathogens around the globe, as explored in more detail in the section by Rim et al. below. More studies will be needed to focus on the introgression of quantitative resistance from wild to cultivated species to improve the durability of resistance to various diseases and pests.

#### De novo domestication for resilience to climate change

Despite several wild relatives having remarkable tolerance to biotic and abiotic stresses, successful introgression of these traits into elite backgrounds has been difficult due to linkage drag (Nevo and Chen, 2010) and the complexity of most traits. De novo domestication, the incorporation of domesticated genes into the nondomesticated species to develop new crops (Razzaq et al., 2021), presents a novel opportunity for immediate utilization of the novel resilience alleles in CWRs. The availability of vast genomic and phenomic resources allow for machine learning (Niazian and Niedbała, 2020) and more precise genome editing (Hua et al., 2019). An excellent example of de novo domestication has been reported in *Solanum pimpinellifolium* (Zsögön et al., 2018). There are now several countries that have exempted genome-edited plants from genetically modified organism regulations, making it possible to utilize de novo domesticated plants as soon as they are generated.

### Development of disease-resistant crops for a changing climate

#### (By Ellen Youngsoo Rim, Alexandra M. Shigenaga, Pamela C. Ronald)

Plant reactions to a single stress differs from those of plants exposed to combined abiotic and biotic stresses, with a shift in signaling pathways and transcriptomic responses (Atkinson and Urwin, 2012; Prasch and Sonnewald, 2013; Sharma et al., 2013). Thus, understanding how plants respond to pathogen stress under nonoptimal environmental conditions is essential for the development of resilient crops in a changing climate (Chaloner et al., 2021; Velásquez et al., 2018).

Diverse plant–pathogen interactions have been shown to be affected by adverse environmental conditions, leading to increased host susceptibility, or in some cases, increased host resistance (Velásquez et al., 2018). Various plant species become more susceptible to fungal, viral, or bacterial pathogens in response to elevated temperatures (Cohen and Leach, 2020; Velásquez et al., 2018). For example, exposure to elevated temperature combined with drought stress led to greater susceptibility to *Turnip mosaic virus* due to downregulated defense response gene expression (Prasch and Sonnewald, 2013). However, there are examples where high temperatures led to enhanced host resistance against pathogen pressure (Venkatesh and Kang, 2019). Similarly, the impact of increased atmospheric CO_2_ in relation to plant–pathogen interactions remains unsettled. For example, high CO_2_ concentrations led to increased susceptibility of wheat to fungal infection (Váry et al., 2015), whereas soybean (*Glycine max*) exhibited either enhanced or reduced susceptibility to infection, depending on the pathogen studied (Eastburn et al., 2010). Deeper insight into the complex interplay among abiotic and biotic stresses will inform ongoing work to mitigate crop damage caused by extreme climate conditions or pathogens.

One mitigation strategy is the application of beneficial microbes that enhance plant health and immunity. For instance, Actinobacteria in the genus *Streptomyces* are enriched in the root microbiome of plants under drought stress (Naylor et al., 2017). Application of *Streptomyces* strains to seeds improved wheat growth and yield in drought field conditions (Yandigeri et al., 2012). Beneficial strains of *Trichoderma* fungus heightened plant immunity and antagonized pathogenic fungi (Tyśkiewicz et al., 2022). Soil application of *Trichoderma* reduced fungal infection in soybeans, tomatoes, peanuts (*Arachis hypogaea*), and other crops (Zin and Badaluddin, 2020). Inoculation with *Trichoderma* has also been shown to enhance tolerance to abiotic stresses such as drought and salinity (Zhang et al., 2016; Scudeletti et al., 2021). Biocontrol strategies present an opportunity to enhance resilience to environmental stress and disease pressure, while reducing the use of chemical pesticides or fertilizers that can further damage the environment.

**Figure 8:**
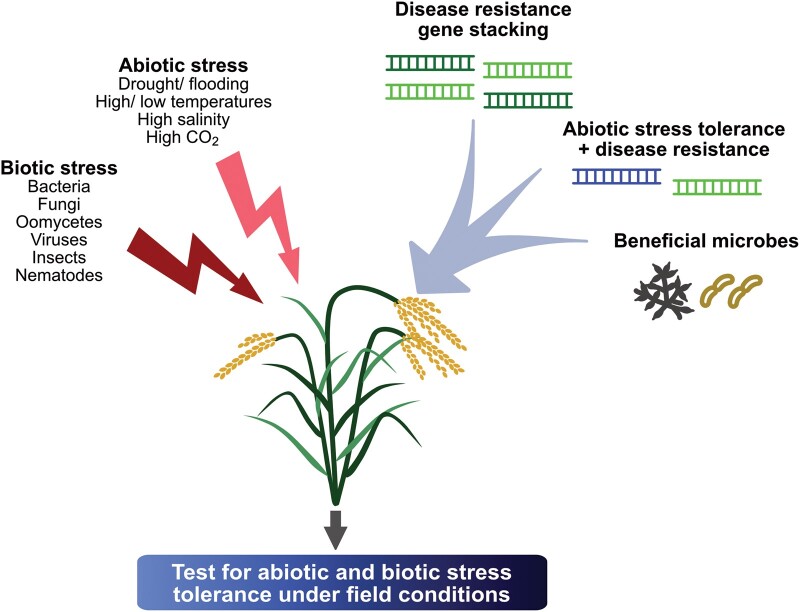
Development of crops with enhanced resilience to abiotic and biotic stress. Crops are exposed to a variety of stresses. Abiotic stresses will intensify as the following climate conditions change: water availability, precipitation, temperatures, and atmospheric CO_2_ levels. Biotic stressors that plants encounter will vary, but may consist of: bacteria, fungi, oomycetes, nematodes, viruses, and insect pests. As climate change alters environmental conditions and plant-pathogen interactions, strategies to develop more climate-ready and disease-resistant crop varieties include breeding or genome engineering approaches with stacking disease resistance genes, stacking climate tolerance and disease resistance genes, and/or addition of beneficial microbes (see text for examples).

Another strategy well-aligned with sustainable agriculture practices is the introduction of genetic improvements that protect crops against pathogens and confer resilience to abiotic stress in a heritable manner. There are numerous examples of genetic alterations in crops leading to resistance to specific pathogens, many of which were achieved through the introduction of immune receptors (Ercoli et al., 2022). Immune receptors are activated by direct or indirect interaction with microbial molecules to elicit host defense responses. Advances in genome sequencing and analysis have accelerated the discovery of immune receptors and other beneficial genetic traits in cultivated crop varieties and their wild relatives (Ercoli et al., 2022; Zsögön et al., 2022; and discussed in the section above by Odeny). For instance, a previously unknown variant of the immune receptor FLS2 was identified in the genome of a wild grape species (*Vitis riparia*; Fürst et al., 2020). The introduction of the new FLS2 variant conferred resistance to *Agrobacterium tumefaciens* in tobacco, offering a potential strategy to control crown gall disease, which affects many crops including nut trees and grapevines. Once identified, desirable genetic traits can be introduced into crops through methods such as marker-assisted breeding or genetic engineering. The use of gene-stacking to introduce multiple protective genes into a single background will likely be an important consideration in engineering climate resilience (Figure 8).

Genome engineering tools such as CRISPR–Cas9 and transcription activator-like effector nucleases allow greater control over the sequence and genomic location of these genetic changes. Crop engineering is an especially promising avenue for mitigating vector-borne plant diseases, which are anticipated to rise as higher temperatures expand the geographical distribution and survival of insect pests (Perilla-Henao and Casteel, 2016; Huang et al., 2020; Skendžić et al., 2021). For instance, introduction of proteins that target the insect vector or the pathogen itself can confer host resistance. Expression of antimicrobial proteins that bind the membrane of *Xylella fastidiosa*, the causative agent of Pierce’s Disease, decreased disease incidence in grapevines (*Vitis vinifera*; Dandekar et al., 2019). Such genetic strategies may lead to more effective and sustainable management of vector-borne diseases, which have relied heavily on chemical insecticides.

Introduction of beneficial traits, however, have mainly focused on the development of crop varieties with resistance to a single stress. Major hurdles remain in engineering crops with combined stress tolerance (Steinwand and Ronald, 2020). For one, enhanced stress tolerance is often accompanied by fitness costs, such as reduced plant growth and yields (Velásquez et al., 2018; Venkatesh and Kang, 2019). Additional genetic interventions can reduce detrimental effects of overactive defenses. For instance, growth penalties associated with powdery mildew resistance in wheat were reversed upon ectopic activation of genes encoding sugar transporters through a mechanism yet to be elucidated (Li et al., 2022). In another example, necrosis associated with broad spectrum potato virus resistance was eliminated through mutating the regulatory region of the resistance-conferring immune receptor (Harris et al., 2013). Alternatively, protective genes can be designed to be expressed under specific conditions. High temperatures increase susceptibility of the model plant Arabidopsis to the bacterial pathogen *Pseudomonas syringae* pv. *tomato DC3000* (*Pst*; Wang et al., 2009; Huot et al., 2017). Disease resistance genes expressed under a heat-inducible promoter protected against *Pst* infection after exposure to high temperature without pleiotropic growth defects (Leng et al., 2021). Another challenge is that pathogens can overcome resistant traits by developing novel virulence strategies or by evolving mechanisms to evade detection by existing immune receptors. Gene stacking might be used to delay or prevent the evolution of resistance-breaking pathogens under diverse climate stresses.

A promising avenue to simultaneously reduce crop loss to pathogen and environmental stress is introducing disease resistance in the context of climate resilience (Rivero et al., 2022; Figure 8). Identification of resistance genes that are more effective under abiotic stress, such as increased temperature, is one approach (Chen et al., 2018; Dossa et al., 2020). For example, stacking the rice disease resistance genes *Xa4* and *Xa7* provided enhanced resistance to *Xanthomonas oryzae* pv. *oryzae (Xoo)*, the causal agent of bacterial blight disease, under high temperature conditions (Dossa et al., 2020). Alternatively, disease resistance genes can be introduced into crop varieties that already sustain high resilience to abiotic stress. The introduction of six genes associated with resistance to the fungal pathogen *Magnaporthe oryzae*, the causal agent of rice blast disease, into a rice variety with elevated drought tolerance resulted in plants that are both resistant to blast infection and tolerant of drought stress in the field (Carrillo et al., 2021). Another approach is stacking genes, through breeding or genome engineering, to confer both abiotic stress tolerance and disease resistance. For example, submergence (*Sub1*) and salt (*Saltol*) tolerance genes were stacked with eight pathogen and pest resistance genes in an elite rice line (Das and Rao, 2015); this line showed resistance to *M. oryzae*, *Xoo* and gall midge, as well as tolerance of submergence and salinity. In some cases, genes can act as regulatory hubs to control abiotic and biotic signaling pathways and are particularly valuable candidates to target for crop engineering (Husaini, 2022). For example, the rice transcription factor MADS26 orchestrates abiotic and biotic stress responses. Downregulation of *MADS26* led to enhanced resistance to *M. oryzae* and *Xoo* as well as drought tolerance in the field (Khong et al., 2015). The effectiveness of each of these strategies to develop resilience to multiple types of stress will vary depending on the crop, pathogen, and environmental conditions. Research on various strategies and their evaluation under field conditions will be crucial to combat the negative effects of climate change on agricultural systems.

### Mycorrhizal and rhizobial symbioses under climate change challenges

#### (By Xiaowei Zhang, Ertao Wang)

**Figure 9:**
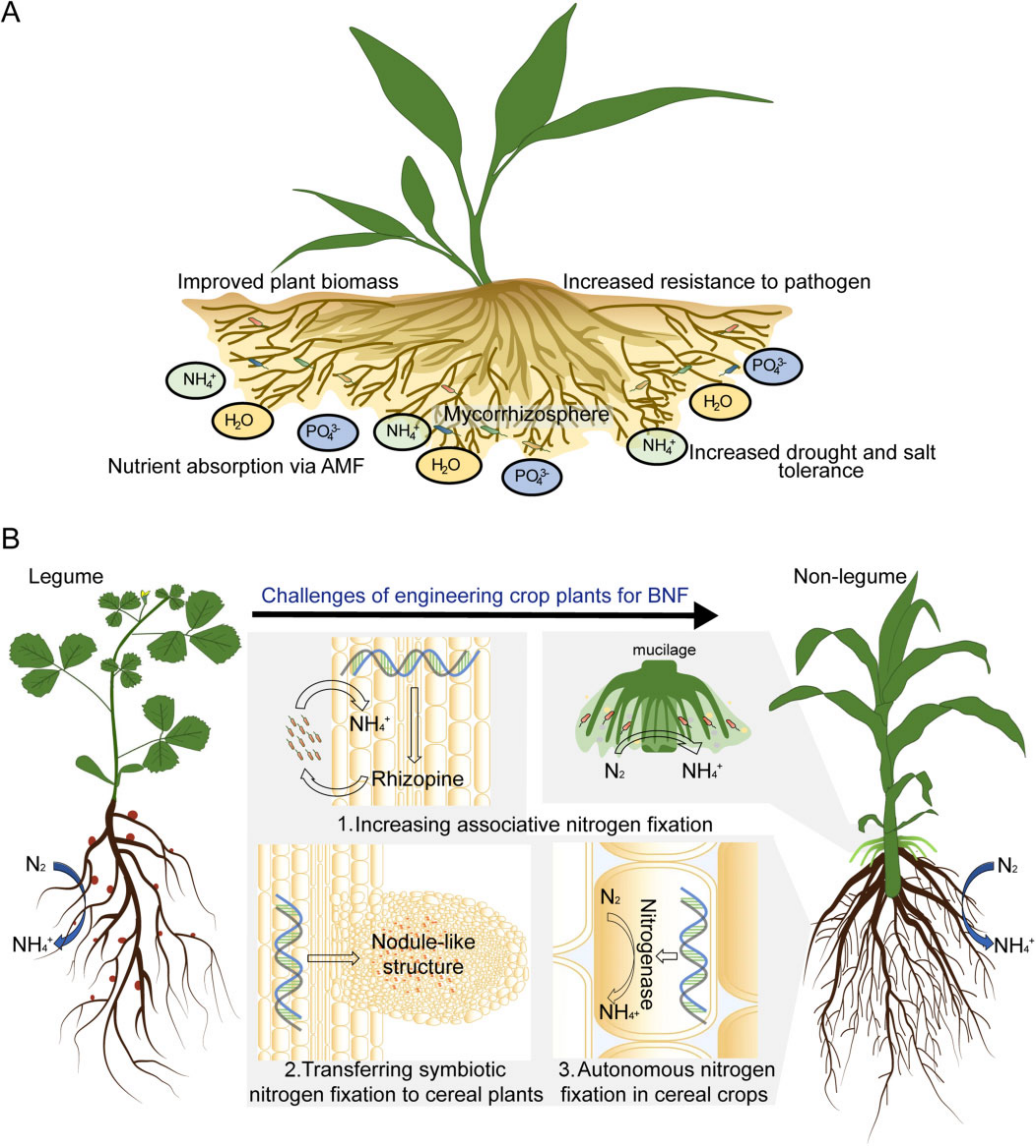
Mycorrhizal symbiosis and N self-fertilizing crops. A, Positive effects of mycorrhizal symbiosis. The mycorrhizal hyphal network forms a mycorrhizosphere (light brown) which can enlarge the plant nutrient absorption area and supply a convenient zone for root-related microbes. Benefits from mycorrhizal symbiosis include increased tolerance or resistance to abiotic or biotic stresses. B, Three steps to develop N self-fertilizing cereal crops to enhance climate change resilience. (1) Increasing associative N fixation. The mucilage (light green) is rich in carbohydrates and harbors abundant diazotrophic microbiota (pink). Engineered cereal plants (such as maize) have the ability to produce rhizophine, which can be perceived by engineered diazotrophs (orange). (2) Transferring symbiotic N fixation to cereal plants. Cereal crops are engineered for symbiotic N fixation by expressing the chimeric receptors perceiving rhizobia signals and overexpressing key symbiotic regulators (*CSSP* genes, *CRE1*, etc.) and nodule development genes (*SCR-SHR*, *LBD16*, etc.) to form nodule-like structures. (3) Autonomous N fixation in cereal crops. The ideal plant which could assimilate N2 into ammonium is created by overexpressing rhizobial N fixation genes in plant cells.

N cycling strongly influences climate change as it is closely correlated to the production of CO_2_, N_2_O, and CH_4_. Currently, crop productivity is highly dependent on fertilizer application, particularly N, which has negative environmental effects through nitrate run-off and release of the potent GHG nitrous oxide. The development of high-yielding, disease-resistant crops can be aided significantly by improving associations with symbiotic microorganisms that enhance nutrient assimilation in the host plant. Here, we summarize the potential application of engineered mycorrhizal and rhizobial symbioses in developing self-fertilizing crops and maintaining sustainable agriculture in the era of global climate change (Figure 9).

#### Mycorrhizal symbiosis

Plant roots are associated with diverse microbes, including bacteria, fungi, and viruses collectively called the rhizosphere microbiome. Among them, mycorrhizal fungi are known to improve plant access to nutrients, particularly phosphorus and N. Two major groups are arbuscular mycorrhizal fungi (AMF), which colonize host roots and are widely distributed in plants, and ectomycorrhizae, mainly associated with trees and shrubs (Genre et al., 2020). The soil region influenced by mycorrhizal roots is called the mycorrhizosphere (Priyadharsini et al., 2016), where mycorrhizal fungi sequester carbon and form aggregate particles in soil that have a major impact on the composition of microbial and plant communities (Priyadharsini et al., 2016; Wang et al., 2021). Under a warmer climate, mycorrhizal fungi can increase carbon sequestration by influencing the root/shoot ratio (Zhou et al., 2022). Ectomycorrhizal fungi can significantly affect the carbon sequestration capacity of certain soils, for example in boreal forests (Clemmensen et al., 2015; Genre et al., 2020). Colonization by AMF can mitigate adverse effects of drought and salt stress by improving nutrient uptake, minimizing oxidative damage, and increasing osmotic adjustment (Hameed et al., 2014; Klinsukon et al., 2021).

Several challenges restrict the application of AMF in agriculture as a biofertilizer. Plants vary widely in response to individual mycorrhizal fungi (Klironomos, 2003), and results of research focusing on one or few AMF species under controlled conditions may not translate to field conditions. In addition, many aspects of the signaling and nutrient exchange pathways are shared between mycorrhizal fungi and biotrophic pathogens (Wang et al., 2012; Zhang et al., 2015; Jacott et al., 2017; Jiang et al., 2017; Zhang et al., 2021a). Thus, it is critical to dissect how plants distinguish between AMF- and pathogen-influenced agronomic traits and engage productively with symbiotic microorganisms while simultaneously restricting pathogens.

Mycorrhizal fungi strongly influence host plant phosphorus acquisition. Interestingly, the phosphate starvation response was found to be a core regulator in both a direct phosphate uptake pathway via root hairs and epidermis and an indirect phosphate uptake pathway via mycorrhizal symbiosis (Shi et al., 2021), suggesting the possibility of developing crops that use phosphorus and N more efficiently by coordination of the direct phosphate uptake pathway and mycorrhizal pathway in future.

#### N fixation in legumes and nonlegumes

Biological N fixation is the process by which nitrogenase (an enzyme found only in certain prokaryotes known as diazotrophs) converts dinitrogen gas from the atmosphere into ammonia and is the main path for the formation of combined N in nature. Three forms of N fixation are found in nature: free-living, associative, and symbiotic (Soumare et al., 2020). In associative N fixation, N-fixing microorganisms living on the surfaces or in the interstitial spaces of the plant host use photosynthetic products from the plant as carbon sources to fix N for their own use and provide the excess fixed N to the host (Soumare et al., 2020). In symbiotic N fixation, N-fixing bacteria colonize the cells of plant organs such as root nodules and supply N to support host growth and development, in systems such as Rhizobium/legume, Frankia/alder, and Cyanobacteria/Australian cycads (Pankievicz et al., 2019; Soumare et al., 2020). The rhizobium–legume symbiosis is the most important N fixation system in terrestrial communities.

Three mechanisms have been proposed to develop N self-fertilizing cereal crops to enhance climate change resilience (Figure 9B):

##### 1. Increasing associative N fixation

Diazotrophs are present in the carbon-enriched mucilage in maize aerial roots and were found to contribute 29%–82% of the N nutrition of Sierra Mixe maize in a 5-year field experiment (Van Deynze et al., 2018). Engineering the cereal host and/or the diazotrophs to enhance this association is therefore a promising avenue to increase biologically fixed N in crops.

It has been shown that the engineered expression in *Medicago truncatula* and barley (*Hordeum vulgare*) of rhizopine, a small molecule compound synthesized by a few rhizobia, could be sensed by engineered bacteria *Azorhizobium caulinodans* ORS571 with a 10^3^-fold increase in perception sensitivity (Geddes et al., 2019; Haskett et al., 2022). This provides the possibility of increasing N fixation from endophytic and free-living bacteria associated with crop plants, although the in situ nitrogenase activity was suboptimal. Further experiments should explore optimizing the expression levels of rhizopine biosynthetic genes to reduce fitness costs in host plants due to excessive gene expression.

##### 2. Transferring symbiotic N fixation to cereal plants

The association of legumes with N-fixing bacteria requires several molecular processes common to the mycorrhizal associations that are more widespread in plants, showing that the evolution of the Rhizobium–legume symbiosis utilized many existing processes that facilitate mycorrhizal interactions (Roy et al., 2020; Wang et al., 2022). This close relationship provides a possibility to engineer symbiotic N fixation into non-legume cereal crops by synthetic biology (Mus et al., 2016). Some progress has been made toward this goal: (i) The overexpression of chimeric receptors, for which the extracellular domains of the rice Myc factor receptors MYC FACTOR RECEPTOR1 (OsMYR1) and CHITIN ELICITOR RECEPTOR KINASE1 (OsCERK1) were replaced with those from the *M. truncatula* Nod factor receptors NOD FACTOR PERCEPTION (MtNFP) and RECEPTOR-LIKE KINASE3 (MtLYK3), respectively, triggers calcium spiking in response to a low concentration Nod factor treatment in rice (He et al., 2019). (ii) The overexpression of several symbiotic regulators induces spontaneous root-nodule-like structures (Soyano et al., 2013; Tirichine et al., 2007; Yang et al., 2022). (iii) The key development genes in *M. truncatula SHORT ROOT* (*MtSHR*), *SCARECROW* (*MtSCR*), and *LLOB-DOMAIN PROTEIN16* (*MtLBD16*) specify cortical cell fate with the ability to de-differentiate to form nodule primordia in response to symbiotic signals (Schiessl et al., 2019; Soyano et al., 2019; Dong et al., 2021). This constitutes a genetic toolkit to generate nodule-like structures to accommodate N-fixing rhizobia, that is by engineering the expression of these key regulators of nodule organogenesis in cereal crops. However, creating the micro-aerobic conditions necessary for rhizobia in the nodule organs of cereal crops to perform N fixation is still a black box.

##### 3. Autonomous N fixation in cereal crops

An ideal approach for self-fertilizing cereal crops would be to make them fix N autonomously. A detailed study showed that the smallest N fixation operon consists of 9 genes, *nifB*, *nifH*, *nifD*, *nifK*, *nifE*, *nifN*, *nifX*, *hesA*, and *nifV* in *Paenibacillus* WLY78 (Wang et al., 2013b). Transgenic Arabidopsis expressing a nine-*nif* gene cassette (*nifBHDKENXhesAnifV*) showed moderate nitrogenase activity and resulted in higher biomass and chlorophyll compared to control plants grown in low-N or N-free medium (Yao et al., 2021). If the results of this study can be validated, this will provide the possibility to construct cereal crops capable of autonomous N fixation in the future.

### Enhancing climate resilience in the hardy staple crop cassava

#### (By Marnin Wolfe, Eder Jorge de Oliveira, and Ismail Rabbi)

Cassava (*Manihot esculenta*) is a staple root crop grown on more than 28 million hectares and crucial to the food security of almost half a billion people. Cassava is uniquely positioned as one of the most climate change resilient crops due to its ability to tolerate prolonged droughts, often exceeding 5 months. The cultivation of cassava has continued to increase in tropical regions, where climate change impacts will be particularly adverse (El-Sharkawy, 1993; Parry and Rosenzweig, 1993; de Oliveira Aparecido et al., 2020). In this section, we overview the innovations that have recently accelerated cassava genetic improvement, the challenges that drought and heat are expected to pose in coming decades, and address prospects to improve climate resilience through interdisciplinary innovations.

#### The NextGen cassava breeding project: A decade of innovation

Cassava is a clonally propagated crop domesticated in South America that continues to radiate throughout the tropics. Phenotypic recurrent selection has been the mainstay of cassava breeding in much of its history. As a result of its 12- to 24-month growth cycle, low multiplication rate and low-seed set, phenotypic selection requires 4–6 years between crosses, a major bottleneck for genetic improvement (Ceballos et al., 2015). Cassava has emerged as a model for the adoption of new breeding technologies among root and tuber crops, including the incorporation of improved experimental designs and phenotyping, as well marker-assisted selection (Mbanjo et al., 2021).

In 2012, the Next-Generation Cassava (NGC) Breeding Project initiated a multi-disciplinary effort to accelerate genetic improvement, notably using genomic selection at breeding programs in Africa and Latin America. NGC partners in Africa include the International Institute of Tropical Agriculture and the National Root Crops Research Institute in Nigeria; the West Africa Center for Crop Improvement in Ghana; the National Crops Resources Research Institute, Uganda, and Makerere University in Uganda; and the Tanzania Agricultural Research Institute in Tanzania. In South America, collaborators include EMBRAPA in Brazil and the International Center for Tropical Agriculture in Colombia. In the USA, collaborators are Cornell University, the Boyce Thompson Institute at Cornell, the University of Hawaii, and the USDA-ARS in Ithaca, NY. The details of partners and funding found at https://www.nextgencassava.org.

**Figure 10:**
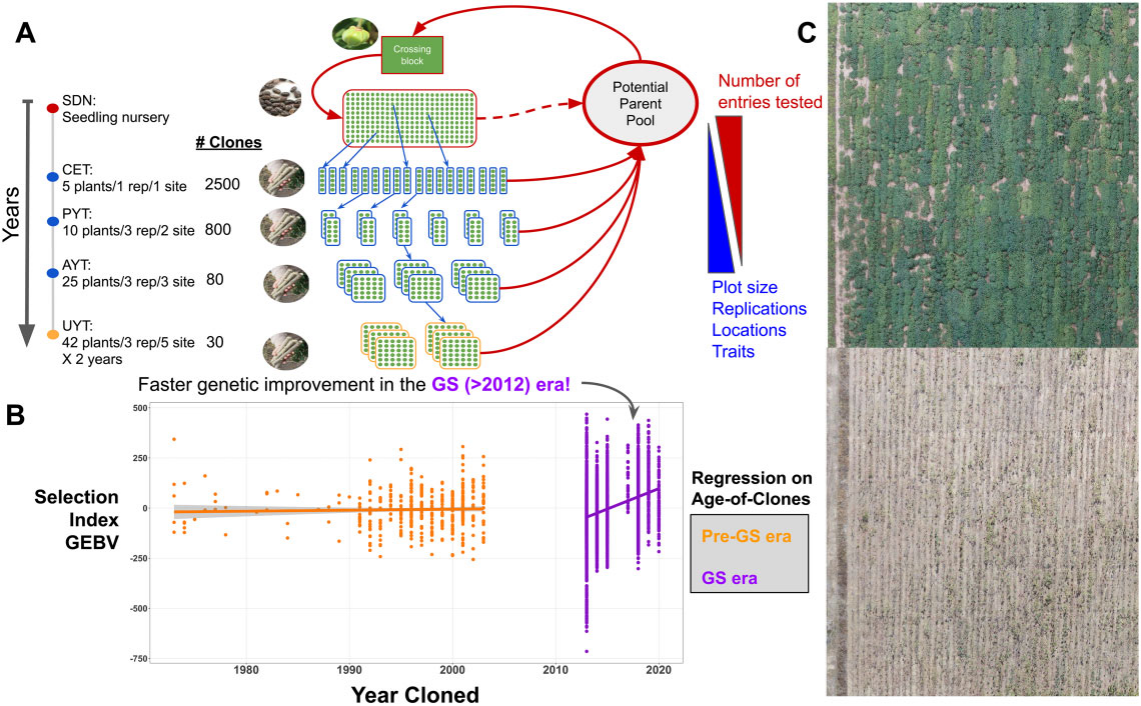
Genomic selection (GS) in a cassava breeding program. A, Each breeding cycle begins with a crossing block trial where seeds are generated. The first evaluation, a seedling nursery (SDN) usually involves >10K plants, but cassava does not produce storage roots when planted from seed and no yield data is collected. After 12 months, seedlings are cloned (5–10 cuttings/plant) into their first single-row, unreplicated clonal evaluation trial (CET) followed by at least three stages of yield trials (preliminary [PYT], advanced [AYT], and uniform [UYT]). All lines entering CET are genotyped genome-wide; sometimes this is done during the seedling nursery. As a result, genomic prediction enables selection of new parents for crossing even as early as the SDN (dashed red arrow). B, GS has resulted in demonstrable acceleration in the rate of genetic improvement since initiation in 2012. Results shown are from the IITA GS population. The genomically predicted performance of GS-era (purple) and historical (yellow) clones relative to a multi-trait selection index (*y*-axis) is plotted against the year when each clone was first generated (*x*-axis). C, Field trial showing variability for one of the major future challenges to cassava: drought. The top image shows plants 3 months after planting, under irrigation at Petrolina (Pernambuco, Brazil). The bottom image shows plants 3 months later under water deficit.

Instead of requiring phenotyping breeding lines over many years before selecting new parents, genomic selection enables breeders to predict performance based on genome-wide genetic markers, even at the seedling stage (Figure 10A). Genotyping of all germplasm and targeted phenotyping of representative subsets make breeding value prediction in early stages possible, increasing selection intensity and reducing selection cycle time (Heffner et al., 2009). The transition from phenotypic to genomic selection has gained momentum in cassava through the NGC, and while breeding cycle times are 50% shorter, selection intensity and accuracy are higher (Wolfe et al., 2017), and the rate of improvement is demonstrably increased relative to previous decades (Figure 10B**)**.

Several additional innovations came to cassava under NGC, including: (1) GWAS enabling the cataloging and validation of trait-linked single-nucleotide polymorphisms used for marker-assisted selection (Wolfe et al., 2017; Zhang et al., 2018; Rabbi et al., 2020); (2) Cassavabase.org, an open-access, breeding database for efficient management of phenotype and genotype data (Morales et al., 2022); and (3) use of plant growth regulators for improved flowering and seed set (Hyde et al., 2020). Genomic resources developed during the last decade, including reference genomes (Lyons et al., 2021) and HapMap (Ramu et al., 2017; Kuon et al., 2019) have laid a foundation for trait-discovery research. These technologies collectively will enable breeders worldwide to tackle the food-security challenges posed by climate change.

#### Climate resilient cassava breeding: Innovations for the next decade

Although cassava is considered a drought-tolerant species (Okogbenin et al., 2013), there is still a large gap between the yield obtained by farmers in semi-arid regions (9.5 t.ha^−1^) and yield observed under experimental water deficit (23.6 t.ha^−1^) with improved genotypes (de Oliveira et al., 2015). Fortunately, there is enormous genetic variability to tap for drought tolerance for future genetic improvement (de Oliveira et al., 2017; Figure 10C). Most cassava field testing by breeders is done in both high-rainfall and drought-prone environments. Over the annual cropping cycle, genotypes are routinely exposed to 3–5 months of drought and higher temperatures during which they are evaluated for leaf retention, greenness, and damage by dry season pests such as green mites (Ezenwaka et al., 2018). Advanced testing is usually done in multienvironment trials, including low rainfall, heat-stressed environments (Hershey, 1984). Although genetic control of drought tolerance in cassava, as measured by yield under drought, is complex with strong genotype-by-environment interaction (de Oliveira et al., 2015), a recent GWAS identified candidate genes with known association to drought tolerance and markers useful for breeding (dos Santos Silva et al., 2021).

The complexity of drought-tolerance genetic architecture suggests that genomic selection, augmented by genome editing and cutting-edge phenomics, will be necessary for the rapid development of climate-resilient cassava varieties. Currently, final yield is the basis for selection for drought tolerance (Khadka et al., 2020). However, yield is affected by many factors into drought and is only measurable after 10–12 months. Earlier stage, nondestructive evaluation of physiological drought responses and root bulking is needed. Remote sensing of photosynthetic performance using drones with hyperspectral imaging (Verma et al., 1993; Banerjee et al., 2020) and root yield using ground penetrating radar is now possible (Agbona et al., 2021). High-throughput phenotyping plus genomic prediction and GWAS-based discovery are powerful tools for climate-resilience breeding (Juliana et al., 2019; Jha et al., 2020). Pilot tests have been conducted on cassava for association with above and below-ground traits (Selvaraj et al., 2020).

Genome editing and metabolic engineering are promising supplements to exploiting existing natural diversity. Transformation of cassava to express isopentenyl transferase resulted in increased water retention and leaf retention under water stress (Zhang et al., 2010). The overexpression of transcription factors like DEHYDRATION-RESPONSIVE ELEMENT BINDING PROTEIN (DREB), ABA-RESPONSIVE ELEMENT BINDING PROTEIN1 (AREB1), and ABA-RESPONSIVE ELEMENT BINDING FACTOR2 (ABF2) were also shown to increase drought tolerance in some species (Rivero et al., 2007). In Arabidopsis, CRISPR/Cas9 was used to modify the *OPEN STOMATA2* (*OST2*) gene resulting in greater drought tolerance through enhanced stomatal response (Osakabe et al., 2016). Engineering multiple traits such as improving light reaction efficiency, reducing photorespiration, improving sucrose synthesis to increase sucrose loading and stimulate cambium activity could improve starch synthesis and metabolite transport into storage roots and increase sink capacity (Obata et al., 2020; Sonnewald et al., 2020). In cassava, there are no published studies on the use of genome editing to mitigate drought response, but several studies have demonstrated the feasibility of using CRISPR/Cas9 for virus resistance (Gomez et al., 2019) and reducing cyanogenic compounds (Gomez et al., 2021).

Climate change will disproportionately impact already food insecure regions of the world (Easterling and Apps, 2005). Cassava, already a hardy crop, can help to attenuate some of those negative impacts. For example, cassava planted under free-air CO_2_ enrichment has been shown to positively respond with increased yield and higher WUE (Rosenthal et al., 2012; Ruiz-Vera et al., 2020). Given sufficient investment, the role of cassava as a food-security and industrial crop will continue to expand and serve as a buffer to future climate change-related food insecurity. We have described ways in which cassava is a “climate-smart” crop and an important staple for millions in the tropics. Now is the time to continue the modernization in cassava breeding and biotechnology to benefit the most vulnerable populations.

### The carbon nutrient penalty: Will it matter?

#### (By Gabriel Castrillo, Martin R. Broadley, and David E. Salt)

Hidden hunger, the lack of sufficient dietary micronutrients including iron (Fe) and zinc (Zn), is a major problem for a significant portion of the world’s human population (Kumssa et al., 2015; Lenaerts and Demont, 2021). Experiments with plants cultivated in growth chambers have suggested that elevated atmospheric CO_2_ is associated with a decline in mineral nutrients in a number of crops, for example, decreased Fe and Zn concentrations in wheat, barley, and rice (Manderscheid et al., 1995; Fangmeier et al., 1997; Seneweera and Conroy, 1997; la Puente de et al., 2000; Pleijel et al., 2000). Free-air CO_2_ enrichment (FACE) experiments with plants grown under standard field management practices, with various crops including soybean, sorghum (*Sorghum bicolor*), potatoes, wheat, barley, and rice (Prior et al., 2008; Högy and Fangmeier, 2009; Högy et al., 2009; Erbs et al., 2010; Fernando et al., 2014a, 2014b; Ujiie et al., 2019), showed similar decreases in mineral nutrients. A more comprehensive set of FACE experiments were reported across three countries, with multiple sites and crops (Myers et al., 2014, Dietterich et al., 2015), which confirmed decreases in Zn and Fe concentration of 5%–10% for C_3_ grains and legumes at the elevated CO_2_ concentrations predicted for 2050 (546–586 ppm). A large meta-analysis representing numerous FACE and non-FACE experiments also identified similar reductions in Zn, and in other dietary mineral nutrients such as calcium (Ca) and magnesium (Mg; Loladze, 2014). This carbon nutrient penalty was projected to cause a decrease in the global availability of dietary Fe and Zn of between 2.5% and 3.6% by 2050 (Beach et al., 2019); producing the forecast that many countries that currently have high levels of hidden hunger will continue to do so.

A better understanding of the impact of elevated CO_2_ on mineral nutrient concentrations in crops requires concomitant consideration of elevated temperature, as they go hand in hand. Combined FACE and temperature (T-FACE) experiments have begun to address the possible impact of elevated temperatures on the carbon nutrient penalty. In soybean, elevated CO_2_ caused a decrease in seed Fe and Zn concentrations (as previously observed), while elevated temperature had the opposite effect; but the combined effect of elevated temperature and CO_2_ restored seed Fe and Zn concentrations (Köhler et al., 2019). A similar compensating effect of elevated temperature on the carbon nutrient penalty was also observed in rice and wheat (Guo et al., 2022). Under uniform global temperature increases, the carbon nutrient penalty may therefore be expected to disappear. However, rising global temperatures will not be uniform across the globe, with different regions experiencing different levels of warming. Predicting if elevated temperatures will balance nutrient loss due to elevated CO_2_ may be more complex and uncertain.

Improved access to diverse diets, comprising more nutrient-dense foods, can play a role in alleviating hidden hunger. However, access to micronutrient-adequate diets is unlikely for many people in the coming decades, for socioeconomic reasons (Nelson et al., 2018). Geographical constraints to micronutrient availability in many food systems, reported from recent GeoNutrition surveys, further compound this challenge (Gashu et al., 2021). Interventions to alleviate hidden hunger include supplements, food fortification, and biofortification of staple crops through breeding and agronomy. Zn-biofortified wheat varieties released in India and Pakistan (Zia et al., 2020; Govindan et al., 2022), and Zn-biofortified hybrid maize varieties in Guatemala and Colombia (Maqbool and Beshir, 2019) can increase grain Zn concentration by more than the anticipated decreases due to elevated CO_2_. The continued development of crops that can reliably accumulate sufficient quantities of mineral nutrients against a backdrop of climate change is an important part of this solution. The use of micronutrient fertilizers (Joy et al., 2017) and “regenerative” agricultural interventions (Manzeke-Kangara et al., 2021) can also play a role in reducing hidden hunger.

Our understanding of mineral nutrient homeostasis in plants is extensive, with over 176 genes identified to date (Whitt et al., 2020), but far from complete. Of these known genes, over 80 are characterized as ion transporters, many of which were investigated based on their predicted function as transmembrane proteins. High-throughput elemental analysis of plant material, also known as ionomics (Salt et al., 2008), has proved to be a powerful forward genetic screening tool that allows the discovery of genes involved in mineral nutrient homeostasis and the study of natural genetic variation in the system (Huang and Salt, 2016). This approach highlights the critical importance of the Casparian strip in the endodermal cell wall in controlling mineral nutrient homeostasis (Hosmani et al., 2013; Pfister et al., 2014; Kamiya et al., 2015; Reyt et al., 2020, 2021; Alcock et al., 2021). Ionomics has also revealed a global pattern of natural variation in the leaf and seed ionome of Arabidopsis (Campos et al., 2021), and in rice (Pinson et al., 2015), barley (Houston et al., 2020), soybean (Ziegler et al., 2018), common bean (*Phaseolus vulgaris*; Nazir et al., 2022), peanut (Zhang et al., 2019), and wheat (Gardiner et al., 2018). The application of genome-wide association mapping to this variation has led to the identification of genes controlling variation in numerous elements (Baxter et al., 2010; Chao et al., 2012, 2014; Forsberg et al., 2015; Yang et al., 2018). Ecological studies are starting to reveal the adaptive benefit of this variation for coastal populations (Busoms et al., 2018, 2021).

Soil microbiota contributes to the biogeochemical cycling of elements, soil regeneration, and plant and animal growth and productivity (Custódio et al., 2022). In experiments with Arabidopsis, the root microbiome was shown to control differentiation of the endodermis, a diffusion barrier that affects mineral nutrient homeostasis, through the repression of responses to the phytohormone abscisic acid in the root (Salas-González et al., 2021). However, these mechanisms have not been evaluated under future elevated CO_2_ scenarios. Elevated CO_2_ in the short term increases metabolic activity and microbial biomass in the soil, with a concomitant promotion of plant growth and root exudation, conditions that reduce soil N content (Chen et al., 2014; Xiong et al., 2015; Yu et al., 2018a). Thus, in the long-term, elevated CO_2_ is predicted to have a negative impact on the soil carbon cycle, promoting the depletion of easily decomposed carbon and increasing the degradation of mineralized SOC with a net increase in atmospheric CO_2_ (Yang et al., 2019a). Elevated CO_2_ influences microbial enzymatic activities for phosphorus and N cycling but this effect changes depending on the ecosystem (Naylor et al., 2020). We need to understand microbiome stability in diverse ecological contexts, considering spatial resolution, microbial connectivity, and multi-kingdom composition. This will allow us to feed current models with realistic experimental data to predict the impact of climate changes on soil microbial populations and their interactions with plants, helping us to develop microbial-based strategies to alleviate climate change impacts on soil and food production.

## Can we achieve a biomass-based bioeconomy?

###  

#### (By Maureen C. McCann and Nicholas C. Carpita)

Gross domestic product, a measure of economic prosperity, is tightly correlated with energy consumption. Fossil fuels accounted for 80% of global energy resources in 2020. Coal and gas can eventually be displaced by renewable energy from wind and solar, geothermal and hydroelectric, and nuclear energy (U.S. Department of Energy, 2015). Oil, however, provides both liquid transportation fuels and raw materials for the petrochemical industry. As addressing climate change becomes increasingly urgent, we now need to shift from oil derived from long-dead organisms to living organisms that can provide chemicals, fuels, and materials (Carpita and McCann, 2020). In this section, we imagine a biomass-based, circular bioeconomy, enabled by recombinant DNA technologies, with the potential to decouple our prosperity from fossil fuel consumption (National Academies of Science, Engineering, and Medicine, 2020). To succeed, this bioeconomy must be fully rooted in plant biology.

Natural and engineered oil-accumulating plants and microalgae, such as cyanobacteria, are an important source of liquid hydrocarbons for use as fuel components. To address how plants can displace a significant proportion of oil consumption also requires use of the sugars and aromatics derived from plant cell walls (McCann and Carpita, 2015). Electric and hybrid vehicles powered by renewable energy sources are becoming viable long-term options for light ground transportation (U.S. Department of Energy, 2015). However, air, marine, and heavy-duty modes of transportation, which contribute one-third of US transportation GHG emissions, will remain dependent upon energy-dense, liquid-hydrocarbon fuels for decades because of slow fleet turnover: aircraft, for example, have a service lifetime of 25–30 years. Advanced biofuels, fully compatible with existing engines and transportation infrastructure, can include liquid hydrocarbons produced by chemical or enzymatic catalytic conversion of biomass-derived sugars and aromatics (Huber et al., 2003; Wang et al., 2014).

Plant-based biofuels also offer the potential for the production of valuable chemical co-products. Decades of research have overcome the technological barriers to the production of cellulose-derived glucose and, more recently, lignin-derived aromatics. As a result of new deconstruction technologies that preserve aromatic ring structures (Bozell et al., 2011; Labbé et al., 2012; Parsell et al., 2013; Socha et al., 2014), lignin is no longer a major source of biomass recalcitrance. Catalytic depolymerization of lignin has been achieved without decomposition of cellulose or xylan, enabling the concept of the “lignin-first” biorefinery, where aromatic fuel substrates are removed before cellulose and other carbohydrates are processed (Ragauskas et al., 2014; Schutyser et al., 2015; Key and Bozell, 2016; Yang et al., 2019b). To produce hydrocarbon fuels, deoxygenation reactions must proceed to full chemical reduction, but for chemical products, reactions must necessarily be highly selective to preserve desirable functional chemical groups. The petroleum industry produces a handful of platform chemicals from oil, including ethylene, propylene, C4-olefins, benzene, toluene, and xylene, that are oxygenated to make tens of thousands of chemicals (Wang et al., 2014; Parsell et al., 2015). Plants synthesize highly oxygenated polymers, and the chemical moieties in these structures hold tremendous value as useful building blocks for chemical co-products. Pathways that employ lignin-derived aromatics as substrates to replace commodity chemicals have been envisioned using either enzymatic or chemical catalysis (Wellisch et al., 2010; Zakzeski et al., 2010). Controlled fractionation of biomass with downstream catalytic upgrading provides several value-added streams for the major biomass components: xylans to furfural (Vinueza et al., 2015), lignin to aromatics and dicarboxylic acids (Zeng et al., 2015), and cellulose to hydroxymethylfurfural (Hewetson et al., 2016).

The diversity of plant metabolism, natural and engineered, also provides a foundation for engineering biology to create economic value. Living plant cells synthesize between 100,000 and 1 million kinds of molecules (Fang et al., 2019). Making natural or synthetic products directly in plants can take advantage of orders-of-magnitude greater metabolic complexity and potential product yields than can be achieved in microbial chassis organisms. Efficient production of target compounds in plants will require a systems-level understanding of metabolism and constraints, including tradeoffs between carbon fluxes and cellular energy balances.

The structural complexity of plant cell wall components can provide oligomeric and polymeric substrates for materials such as thermosets, thermoplastics, composites, cellulose nanocrystals, and nanofibers. Thermoset materials include epoxy, silicone, and polyurethane. Lignin- and carbohydrate-derived monomers have been incorporated into polymers to create new bio-based materials with improved performance characteristics compared to fossil fuel-derived thermoset materials (Zhao and Abu-Omar, 2015; Jiang et al., 2018; Chen et al., 2019). Poplar fibers have also been directly incorporated into composites with polylactic acid as a replacement for conventional carbon nanofibers that reinforce polymers for large-scale 3D printing applications (Zhao et al., 2019). In contrast to thermosets, thermoplastics can be melted, and some of their monomers may be recycled. The entire pathway to polyhydroxybutyrate was engineered in cotton over 25 years ago (John and Keller, 1996), and more recently in the bioenergy crop switchgrass (*Panicum virgatum*) (Somleva et al., 2008). Routes for the biological synthesis of polyhydroxyurethane have been envisioned (Nohra et al., 2013). When pulped wood particles are treated with acids, cellulose nanocrystals, and cellulose nanofibers are recovered, derivatives of which are used for several kinds of synthetic materials as replacements for plastics (Moon et al., 2011; Zhu et al., 2016).

Maximizing the recovery of biomass carbon into fuels and co-products requires flexible design capabilities to produce cell wall architectures that can be easily and completely deconstructed for current and future conversion processes (McCann and Carpita, 2015). As robust cell wall architectures are integral to plant growth and development, genetic variants that are tailored with regard to biomass quality for conversion processes must not be compromised for yield or sustainability traits in field performance. Major knowledge gaps include how biosynthetic products are integrated into composite structures, how their individual structural complexities contribute to molecular- to macro-scale architectures, and how cell wall architectures might be redesigned for production of high-value products (Carpita and McCann, 2020).

Material use is tightly coupled to energy use, GHG emissions, land and water use, and waste flows. About one-third of global GHG emissions comes from industrial manufacturing. To decarbonize this economic sector will require changing the means of manufacture as well as the nature of material inputs (Chui et al., 2020). We might imagine how to build simplified production systems with the components of plant cells or make biohybrid materials outside an intact organism. We might imagine designing plants to synthesize homopolymers, heteropolymers and composite materials, displacing structural concrete and steel with new materials like superwood (Chen et al., 2020), or developing new-to-nature materials with advantageous properties.

Food security is a paramount concern and there are land use issues to consider in raising crops for nonfood versus food production. Nonfood cash crops, such as—traditionally—cotton (*Gossypium hirsutum*), tobacco, hemp, hops (*Humulus lupulus*), and biofuel feedstocks, to name just a few, can be of considerable value to growers and mitigate financial risk. The diversification of food and nonfood plant products within a single cropping system, or a single crop, coupled with principles of sustainability and climate change resilience, could thus be an advantage toward achieving both food and energy security. The decarbonization of agriculture could include the use of bioenergy crops to displace fossil fuels as a source of hydrogen for ammonia production (Gencer et al., 2020) as well as displacement of fossil fuels for harvesting and drying.

In the USA, an annual sustainable resource of over 1.6 billion tons of lignocellulosic biomass could be considered a strategic carbon reserve (U.S. Department of Energy, 2016). This quantity of biomass represents double the entire annual output of the US agricultural system—grains, fruits, vegetables, hay, and pasture grasses. To double or triple the capacity of the current agricultural system for a biomass-based bioeconomy, additional acres must be brought into production, all crops must be high-yielding, and growers must benefit from diversification of plant products.

## Conclusions

We presented examples of some plant biology-based solutions that we believe show promise toward enhancing terrestrial carbon sequestration and engineering climate resilient crops. Although we addressed several disparate topics, a few overarching conclusions emerge.

### Innovation

Some of the ideas described here may seem far-fetched to today’s readers, but we believe that for our planet to remain inhabitable and sustainable, many of the ideas proposed here—or others like them—will need to be realized, and we will need plant scientists to help achieve them.

### Collaboration

By definition, efforts to mitigate global climate change must be large scale. Plant scientists contributing meaningfully in this arena most likely will be those who seek out effective collaboration—not only with other plant scientists but also with those in other disciplines, including for example agronomy, bioinformatics, data science, engineering, forestry, and soil science. Improved communication and collaboration across disciplines and between academia and industry can also be viewed as a low-tech effort that can have a strong impact. Identifying and seeking out potential collaborators who can link the research to impactful pathways should be a primary goal early in the planning stages of research projects for maximum benefit. Socio-economic and political perspectives will also be crucial in determining which approaches will be adopted and how quickly they will be implemented. Networking, discussion, and collaboration in the socio-political arena and with industry, governmental, and nongovernmental organizations may also be crucial.

### Implementation

Many current practices have substantial potential for mitigating CO_2_ emissions, including reducing food and agricultural waste, shifting to plant-based diets, reducing deforestation coupled with afforestation/reforestation, and restoring coastal wetlands. Some technological solutions also have potential in the shorter term, including direct air capture, biochar, enhanced rock weathering, and bioenergy combined with carbon capture and storage. To date, none of these options has been implemented globally due to cost, timeline, inefficiency, lack of scalability, or an uncertain and evolving carbon price and market. Estimates for the potential of available technologies vary widely and will depend on the ability of nations to realize effective measures (Roe et al., 2019). We have explored ways that plant science can help to tip the balance toward enhanced climate change mitigation and crop resilience. The section on enhancing carbon capture and sequestration in annual cropping systems speaks to our immediate needs for carbon capture on a massive scale and the possibility that plant scientists can achieve a meaningful impact in this arena.

Some of the goals of examples discussed may require years to realize fully, such as engineering C_4_ photosynthesis into rice, symbiotic N fixation into cereals, and crops that produce a variety of synthetic products. Although time is pressing, this does not make them unworthy of attention. First, aspects of these longer-term goals may provide significant benefits in the short term, and second, the need for carbon capture and enhancing crop resilience and food security will continue in the future. The need is urgent for every plant biologist to consider today how their research can contribute to addressing climate change, ensuring food security, and achieving a sustainable biomass-based bioeconomy.

## Notes

Authors are listed alphabetically (with the exception of the lead author/coordinating editor). All authors contributed to writing and revising the article.

The author responsible for distribution of materials integral to the findings presented in this article in accordance with the policy described in the Instructions for Authors (https://academic.oup.com/plcell) is: Nancy A. Eckardt (neckardt@aspb.org).
